# Estrogen Induced Regulation of Mucosal‐Associated Invariant T Cells in Asthma

**DOI:** 10.1155/jimr/8201923

**Published:** 2026-03-20

**Authors:** Shubhanshi Trivedi, Samuel E. Aamodt, Thomas P. Huecksteadt, Elizabeth J. Myers, Grace Helen McGee, Jackson G. Cacioppo, Jeffrey Aubé, Robert Paine, Daniel T. Leung, Kristi J. Warren

**Affiliations:** ^1^ Department of Internal Medicine, University of Utah, Salt Lake City, Utah, USA, utah.edu; ^2^ George E Wahlen Department of Veterans Affairs Medical Center, Salt Lake City, Utah, USA; ^3^ Department of Pathology, University of Utah, Salt Lake City, Utah, USA, utah.edu; ^4^ Department of Chemistry, The University of North Carolina at Chapel Hill, Chapel Hill, North Carolina, USA, unc.edu

**Keywords:** asthma, mucosal-associated invariant T (MAIT) cells, estrogen (E2), estrogen receptors (ER) α and ERβ, G-protein coupled estrogen receptor (GPER-1), interleukin-2 (IL-2) IL-7 IL-12 IL-15 IL-18

## Abstract

Asthma affects over 300 million people worldwide, with increasing rates annually. Males have higher asthma prevalence in adolescence, while females exhibit higher rates in adulthood. Reduced mucosal‐associated invariant T (MAIT) cells are found in severe asthmatic airway disease. We hypothesize MAIT cells modulate airway inflammation and are regulated by sex hormones through their cognate hormone receptors. We compared circulating MAIT cells and ex vivo MAIT cell activation with MR‐1 ligand in controls and asthmatic individuals. MAIT cells were significantly lower in asthmatic males and females compared to healthy controls. MAIT cells derived from male and female asthma patients exhibited higher levels of estrogen receptors (ERs) than those from sex‐matched healthy controls, and ex vivo treatment with estrogen significantly decreased IFN‐γ production in asthmatics. Estrogen treatment did not reduce IFN‐γ in MAIT cells from healthy individuals. To explore the effect of estrogen on MAIT cells, we used the murine *Alternaria alternata* challenge model. Adoptive transfer of G‐protein coupled ER (GPER‐1) antagonist (G36)‐treated MAIT cells into Rag1 knockout mice (Rag1^−/−^) increased *A. alternata*‐induced inflammation compared to those receiving MAIT cells without GPER‐1 blockade. These findings suggest GPER‐1 as a novel target to reduce airway disease in the asthmatic population.

## 1. Introduction

Asthma is a chronic airway disease affecting over 300 million people worldwide [1]. Age and biological sex are important factors influencing asthma development and severity. In adolescence, boys have a higher prevalence of asthma than girls, and in adulthood, women have higher rates and increased severity of asthma as compared to men [2]. Sex hormones are key mediators of this transition, from childhood to adulthood, in the prevalence of asthma between the biological sexes [3–7]. 30%–40% of asthmatic women report worsening of asthma symptoms during the perimenstrual phase [8], and the fluctuations of estrogen and progesterone are the likely culprits for this effect [9, 10]. Among postmenopausal women, hormone replacement therapy has been associated with reduced asthma exacerbations and improvements in pulmonary function [11–14]. Among adult women hormonal contraceptives reduced asthma exacerbations and wheezing [6, 7, 15–17], and large population‐based studies show that stabilizing ovarian hormones with estrogen or progestin‐based birth controls may reduce asthma rates [18].

Estrogens have significant effects on innate and adaptive immunity. Multiple immune cells and parenchymal cells express estrogen receptors (ERs) on their surface, including cells closely related to pathology of asthma, such as eosinophils, mast cells, group 2 innate lymphoid cells, and T cells [19–22]. Estrogen‐mediated signaling events can be divided into genomic and nongenomic effects. In genomic signaling, estradiol (17β‐estradiol; E2) acts through two known hormone receptors, ER α and ERβ in the cytoplasm. The complex dimerizes and translocates to the nucleus to induce transcriptional changes in estrogen‐responsive genes with or without estrogen response elements [23–25]. In contrast to genomicsignaling, nongenomic signaling is initiated from the plasma membrane by GPER‐1, that also binds 17β‐estradiol to induce nontranscriptional changes [26]. Some studies report downregulated airway hyperresponsiveness (AHR) upon administration of estrogen in females and OVX mice [27–29]. Others report exacerbated AHR and remodeling in ERβ knockout mice but reduced AHR in ERα knockout mice [30]. In our studies we have found that steady levels of estrogen decreased type 2 inflammation yet increased neutrophilic inflammation and airway resistance in the OVA‐induced allergic inflammation model [31]. Depending upon which ER is activated, these studies highlight the diverse effects of estrogens on the immune response to allergens.

Mucosal‐associated invariant T (MAIT) cells are innate‐like αβ T cells implicated in airway inflammation and immunity [32, 33]. MAIT cells make up 3% of T cells in human airways [34–36], have responses in humans that differ by sex in both healthy and disease states [37, 38], including obesity [39], COVID‐19 [40], ankylosing spondylitis [41] and nonsmall cell lung cancer [42]. MAIT cell frequency negatively correlates with age [43]. The effect of estrogen on MAIT cell function, especially in the lung mucosa of humans, is not known. Until now, two specific natural antigens derived from the riboflavin pathway 5‐(2‐oxopropylideneamino)−6‐D‐ribitylaminouracil (5‐OP‐RU) and 5‐(2‐oxoethylideneamino)−6‐D‐ribitylaminouracil (5‐OE‐RU), have been identified to strongly activate MAIT cells via their TCR when presented by the major histocompatibility complex (MHC) class I–related protein (MR1) [44]. 5‐OP‐RU and 5‐OE‐RU are formed when 5‐amino‐6‐d‐ribitylaminouracil (5‐A‐RU), a key intermediate in microbial and fungal riboflavin biosynthesis, reacts with α‐dicarbonyl compounds, such as glyoxal and methylglyoxal (MeG), formed from mammalian glycolysis or bacterial metabolism [44–46]. Recently, cholic acid 7‐sulfate (CA7S), a sulfated bile acid, as a first endogenous antigen, has been reported to activate MAIT cells in an MR1‐dependent manner [47]. In addition to TCR‐mediated activation, MAIT cells can get activated in a TCR‐independent manner by inflammatory cytokines like IL‐12 and IL‐18 [48–50]. Activation leads to the release of perforin and granzyme B and pro‐inflammatory cytokines, including interferon‐γ (IFN‐γ), TNF‐α, and IL‐17 [51, 52]. Previous studies have shown that MAIT cells may modulate asthmatic responses [53]. In a birth cohort study, MAIT cell frequency at 1 year of age was associated with a decreased risk of asthma by age 7, suggesting that increased MAIT cells early in life could be protective, potentially reflecting immune programing to T helper 1 (Th1) responses [54]. Because of their lack of alloreactivity [55], and the ability to activate these cells exogenously with MR1 ligands and cytokines, they are attractive tools to explore for reducing asthma exacerbations.

A recent study found that the frequency of MAIT cells in blood, sputum, and biopsy specimens was lower in adult asthmatic patients with severe asthma compared with healthy subjects and mild asthmatics [56]. Another study showed that the frequency of circulating MAIT cells in patients with neutrophilic asthma was lower than that of healthy controls, leaving behind predominantly IL‐17A+ MAIT cells, and a loss of IFN‐γ+ MAIT cells in asthmatics [57]. Additionally, the capacity of MAIT cells to produce IFN‐γ has been linked to improved clinical outcomes in allergic rhinitis patients undergoing allergen immunotherapy [58]. In an *Alternaria alternata*‐induced model of allergic airway inflammation using C57BL/6 mice, MAIT cells restricted allergic inflammation by suppressing the function of group 2 innate lymphoid (ILC2) cells, particularly by limiting expression of Th2‐cytokines (IL‐5 and IL‐13) in ILC2 through MAIT cell induced IL4I1[59]. In another study, mice rich in MAIT cells (Vα19 mouse) showed alleviated airway inflammation compared to wild type mice upon challenge with *Alternaria alternata* extract [60], and those MAIT cells similarly restrained ILC2 function via IFN‐γ.

In the present study, we explored sex differences in MAIT cells, and the ex vivo effects of estrogen on MAIT cells isolated from a cohort of asthmatics and healthy controls. Simultaneously, we used the *Alternaria* model to evaluate whether ERs could be targeted to modulate MAIT cell responses. We showed that estrogen suppressed IFN‐γ+ MAIT cells in asthmatic patient samples and found that blocking the estrogen‐GPER‐1 interaction with G36 (a GPER‐1 antagonist) suppressed IFN‐γ production and increased eosinophilic airway inflammation.

## 2. Materials and Methods

### 2.1. Human Subjects

Asthma patients and healthy individuals were recruited from the Salt Lake City VA Medical Center pulmonary clinics and enrolled by the University of Utah Pulmonary Clinical Research Group. Asthma is diagnosed by a clinical history of episodic symptoms with airflow limitation and/or AHR with evidence of airflow obstruction. Patients having any of the following criteria were excluded: (1) Current smokers, e‐cigarette users, or a history of cigarette smoking/e‐cigarette use in the last year. (2) Subjects with any confounding lung diseases, such as chronic obstructive pulmonary disease, interstitial lung diseases, and cystic fibrosis. (3) Female donors were excluded if pregnant, lactating, had undergone oophorectomy, were menopausal or postmenopausal. (4) If subjects were on any immune suppressive medication, such as a systemic steroid, in the 4 weeks prior to blood donation. (5) Subjects with any symptoms of respiratory infection in the last 2 weeks. For immunophenotyping studies, we enrolled male and female asthmatic patients between the ages of 26–55 years, and healthy males and females between the ages of 29–54 years as controls. In total, 24 asthma patients and 30 healthy participants were recruited, demographics data and clinical characteristics described in Table 1. Participants in this study were drawn from a larger parent cohort study [61], and none were using oral contraceptives. After informed consent was obtained, a clinical questionnaire was completed, and whole blood was collected by venipuncture. For hormone receptor and estrogen stimulation experiments, we used de‐identified apheresis cones obtained from blood donors from the ARUP Laboratories Blood Services at the University of Utah. The Institutional Review Boards at the Salt Lake City VA Medical Center and the University of Utah reviewed and approved this study prior to recruitment (IRB #00120600).

**Table 1:** Study participant characteristics.

Characteristic	Healthy	Asthma
Number	30	24
Age, median (range)	43 (29–54)	46 (26–55)
Female	42 (29–54)	47 (26–55)
Male	46 (30–54)	46 (26–53)
Reported sex		
Female (%)	63	46
Male (%)	37	54
Race		
Caucasian (%)	76	83
African American (%)	7	0
Asian (%)	7	0
American Indian or Alaskan Native (%)	0	0
Native Hawaiian or other Pacific Islander (%)	3	0
Other (%)	0	4
Undisclosed/refused (%)	7	13
Ethnicity		
Non‐Hispanic (%)	84	67
Hispanic (%)	13	13
Undisclosed/refused (%)	3	20
Years with asthma (median)	N/A	—
Asthma category		
Allergic asthma	N/A	2
Bronchial asthma	N/A	4
Asthma with shortness of breath	N/A	2
Mild persistent asthma	N/A	2
Moderate persistent asthma	N/A	2
Unspecific asthma	N/A	4
Other asthma	N/A	8

### 2.2. Blood Processing and Cryopreservation

The whole blood was warmed to room temperature (RT) and diluted with phosphate‐buffered saline (PBS) at a 1:1 ratio. PBMC from apheresis cones were isolated by density gradient centrifugation using the Ficoll‐Paque Premium (Cat# 17544203, Cytiva, Uppsala, Sweden) [62]. Blood collected from a participant from pulmonary or VA clinics was isolated using Lymphoprep (Cat# 07851, Stemcell, Canada) in an EasySep‐50 tube; diluted whole blood was overlayed and separated according to the manufacturers protocol, as before [63]. Total PBMCs were counted using the TC‐20 automated counter (Bio‐Rad) with trypan blue exclusion, followed by cryopreservation in 35% DMEM (Cat# D6429, Millipore Sigma, MA), 55% FBS (Cat# A5256701, Thermo Fisher, MA), and 10% DMSO (cat# D2650, Millipore Sigma, MA) media.

### 2.3. Mucosal Associated Invariant T Cell Function and Hormone Receptor Analysis

MAIT cell cytokine production was assessed following 5‐A‐RU stimulation and ER modulation. PBMCs were thawed and washed with phenol red‐free RPMI media supplemented with 10% charcoal, dextran‐stripped FBS (cat# 12676029, Gibco, NY). Cells were preincubated with 1.2 µM G36 (GPER‐1 antagonist, cat# 4759, Bio‐techne, R&D Systems Inc., MN) or 200 nM ICI 182,780 (ER antagonist/GPER‐1 agonist, cat# 1047/1, Bio‐techne, R&D Systems Inc., MN) for 20 min in a 37°C incubator prior to addition of estrogen (cat#50‐28‐2, Millipore Sigma, MA). 1 × 10^6^ PBMC were cultured with 10 pg/mL of estrogen in a round‐bottom 96‐well plate (cat#163320, Thermo Fisher, MA) at 37°C 5% CO2 for 4−5 days in the presence of survival cytokines; human IL‐2 (10 ng/mL; cat# 200‐02‐50UG, Thermo Fisher, MA) and IL‐7 (10 ng/mL; Cat# BT‐007‐010, Bio‐techne, R&D Systems Inc., MN). To model the immunomodulatory effects of estrogen under physiologically relevant low‐dose conditions, we used 10 pg/mL estradiol, a concentration previously employed in other immune studies [64, 65]. About 2 µM 5‐amino‐6‐D‐ribitylaminouracil (5‐A‐RU) and 50 μM/mL Methylglyoxal (MeG; Sigma) was added for 6 h. Brefeldin A Solution (Biolegend) was added at 5 mg/mL for 4 h. Cells were treated with Fixable Viability Dye eFluor 780 (cat# 65‐0865‐14, eBioscience, MA) or Zombie Aqua Fixable Viability Dye (cat# 423101, Biolegend, CA). Cells were quenched with serum‐containing media and incubated with Fc block (cat# 564219, BD Biosciences, NJ) for 15 min at RT, then centrifuged at 300 g for 5 min. Cells were then stained with fluorochrome‐conjugated antibodies: anti‐human CD3 BUV395 (Cat# 563548, BD horizon, NJ), anti‐human CD45 BUV805 (cat# 612891, BD horizon, NJ), anti‐human CD4 BUV737 (cat# 612748, BD Biosciences, NJ), anti‐human CD8 BUV496 (cat# 612942, BD Biosciences, NJ) and anti‐human MR1 5‐OP‐RU Tetramer (National Institutes of Health [NIH] Tetramer Core Facility) for 30 min at RT. To analyze MAIT cell hormone receptor expression, in a separate flow‐antibody panel, PBMC were stained with anti‐human CD3 BUV496 (cat# 612940, BD horizon, NJ), anti‐human CD45 BUV805 (cat# 612891, BD horizon, NJ), anti‐human MR1 5‐OP‐RU Tetramer (NIH Tetramer Core Facility), and anti‐human GPER‐1 (Cat# AF5534, R&D Systems Inc., Minneapolis, MN, USA) conjugated to PE‐Cy7 using PE‐Cy7 conjugation kit (ab102903, abcam, Boston, MA). For intracellular cytokine staining, cells were fixed and permeabilized using a Foxp3/Transcription Factor Staining Buffer set (cat# 00‐5523‐00, eBioscience, MA) and incubated with anti‐human IFN‐γ FITC (cat# 502505, Biolegend, CA) or with anti‐human ERα AF488 (ab19415, abcam, Boston, MA) or anti‐human ERβ FITC (cat# NB156643, Novus Biologicals, Littleton, CO) and progesterone receptor (PR; Dylight 550; cat# NBP233327R, Novus Biologicals, Littleton, CO) for 40 min at RT. Cellular data were acquired on the Cytek Aurora flow cytometer (Cytek Bioscience’s, CA). Positive staining for each marker was compared against the corresponding fluorescence minus one (FMO) control. Flow cytometry data were analyzed using FlowJo v10.

#### 2.3.1. In vitro MAIT cell expansion

PBMCs obtained from apheresis cones from anonymous healthy donors were washed in warm RPMI‐1640, cells were resuspended in expansion medium containing RPMI‐1640 plus 2% Physiologix XF Serum Replacement (Nucleus Biologics) supplemented with 1% penicillin–streptomycin (Gibco), rhIL‐2 (10 ng/mL, PeproTech), and rhIL‐7 (10 ng/mL, PeproTech) [62]. Around 1 × 10^6^ cells were seeded in 1 mL of expansion media per well in a 24‐well plate and cultured for 14 days. 50% of the media was exchanged every 3 days with fresh cytokines, and MAIT cells were stimulated with 10 nM 5‐OP‐RU on Days 0, 5, and 10. Following the expansion, MAIT cells were sorted from culture on Day 14 by FACS. Cells were surface‐stained with eFluor 780 fixable viability dye, anti‐CD3 FITC (BioLegend), anti‐MR1‐5‐OP‐RU PE‐labeled tetramer, anti‐Vα7.2 APC, anti‐CD4 BV785, anti‐CD8 BV605, anti‐CD19 PerCP‐Cy5.5 (BioLegend), and anti‐CD161 AF700 (BioLegend). Vα7.2‐positive cells were collected using a FACSAria Cell Sorter (BD Biosciences) and suspended in RPMI1640 plus 10% FBS and 1% penicillin–streptomycin for downstream use.

### 2.4. RNA and RT–PCR

RNA was isolated from sorted MAIT cells using the Direct‐zol RNA Miniprep kit following the manufacturer’s instructions (Zymo Research #R2050). Samples were run on a PTC‐100 (MJ Research, Inc.) thermo cycler using the High Capacity cDNA reverse transcription kit (Applied Biosystems #4368814) following the kit instructions for cycling conditions. RT‐PCR was performed using TaqMan Universal Master Mix (Applied Biosystems #4304437) and gene expression taqman primers to GPER1, ESR1, and Gapdh (ThermoFisher Scientific). The PCR plate was run on a Quantstudio 12K Flex machine at the University of Utah genomics core facility using the recommended standard TaqMan PCR settings. Relative gene copy number for the gene of interest was determined using the delta–delta CT method.

### 2.5. Immunoprecipitation and Western Blot

After MAIT cells were FACS sorted, protein lysates were made using the cell lysis buffer II (Invitrogen #FNN0021) following included instructions and frozen at −80°C in aliquots. Protein concentration was determined using a DC Protein Assay Kit (Biorad). For the immunoprecipitation, Protein G magnetic beads were used (MedChemExpress, #HY‐K0204). The immunoprecipitation protocol supplied with the magnetic beads was followed. Briefly, 5 μg/mL of GPER antibody (R&D Biosystems #AF5534) was combined with magnetic beads and 0.5% Tween‐20 in PBS and incubated on a tube rotator for 30 min at RT. Magnetic separation was performed, and the antibody–bead complex was added to 20 μg of protein and incubated on the tube rotator for 30 min at RT. After final elution, the sample was added to laemmli buffer and loaded onto a Mini‐Protean TGX gel (Biorad, #4561094) and run with the Mini Trans‐Blot system and power supply from Biorad. The gel was transferred with a Trans‐Blot turbo transfer system for 7 min (Bio‐Rad). The blot was incubated overnight with 1 μg/mL GPER antibody (R&D Biosystems #AF5534) at 4°C. A goat secondary (Licor #926‐32214) was applied for 1 h at RT, and the blot was imaged using a Licor Odyssey CLx system. The blot was quantified using ImageJ.

### 2.6. Cell Lines

The MCF‐7 cell line (*Homo sapiens*, female, breast adenocarcinoma) was used as a control in Western blot experiments in this study. The official cell line name is MCF‐7, and the Research Resource Identifier (RRID) is RRID: CVCL_0031. The cells were obtained from Dr. Hamid Ghandehari, Director of the Utah Center for Nanomedicine at the University of Utah, on March 19, 2025. To our knowledge, this cell line has not been previously reported as misidentified or contaminated. The cell line was confirmed to be free of mycoplasma contamination at the time of use.

### 2.7. Mice, In Vivo Treatments, and Tissue Collection

Six‐ to eight‐week‐old C57BL/6J female mice were acquired from the Jackson Laboratory (Bar Harbor, USA). Rag1 knockout mice (Rag1^−/−^) were initially obtained from The Jackson Laboratory (Bar Harbor, ME) then bred at the University of Utah Comparative Medicine Center. All mice were housed under specific pathogen‐free conditions, and all animal experiments were performed in strict accordance with the NIH Guide for Care and Use of Laboratory Animals. All procedures were reviewed and approved by the Salt Lake City VA Medical Center (ACORP 22‐05) and the University of Utah (IACUC #00001529). Pulmonary MAIT cells were expanded, isolated, and adoptively transferred into Rag1^−/−^ mice according to a protocol described previously [66]. Mice were intranasally inoculated with 16.7 μg Pam2Cys (Invitrogen) or Pam2Cys plus 2 mM 5‐A‐RU plus 50 μM methylglyoxal (Sigma) in 50 μL on Day 0, followed by 1x PBS or 2 mM 5‐ARU/50 μM methylglyoxal on Days 1, 2, and either 3 or 4. On Day 8, mice were euthanized and lungs were perfused with 5 mL of PBS [67]. Single‐cell suspensions were prepared using the gentleMACS Lung Dissociation kit (Miltenyi Biotech) according to the manufacturer’s protocol. Red blood cells were lysed using ACK lysis buffer (Thermo Fisher). Single cell suspensions were stained as described in [67]. Briefly, prior to surface staining, tissue single‐cell suspensions were incubated in Fixable Viability Dye eFluor 780 (Cat# 65‐0865‐14, eBioscience, MA) at RT for 15 min, washed, and incubated in anti‐mouse CD16/CD32 Fc block (Cat# 101301, Biolegend, CA) and unlabeled MR1‐6‐formylpterin‐tetramer (6‐FP; NIH Tetramer core) for 10 min at RT to reduce nonspecific binding. Cells were then incubated for 30 min at RT with mouse PE‐conjugated 5‐OP‐RU MR1‐tetramer (NIH Tetramer core) and the following antibodies: anti‐TCRβ‐BV421 (cat# 109229, Biolegend), anti‐CD3‐BUV395 (cat# 740268, BD Optibuild, NJ), anti‐B220‐PE‐Cy5 (cat# 103209, Biolegend), anti‐CD44‐BV650 (cat# 103049, Biolegend), and sorted using the BD FACS Aria II. MAIT cells were defined as live CD3+ B220− TCRγδ− CD44high TCRβ+ MR1‐Tetramer+ lymphocytes. Approximately 5 × 10^4^–1 × 10^5^ MAIT cells were sorted per mouse. MAIT cells were cultured overnight in the presence of recombinant mouse IL‐12 (10 ng/mL; cat# 577002, Biolegend), IL‐15 (10 ng/mL; cat# 566302, Biolegend), and IL‐18 (10 ng/mL; cat# 767002, Biolegend) with estrogen (10 pg/mL) or with estrogen and G36 (1.2 µM) in phenol‐red free R10 media. On Day 9, 1 × 10^5^ MAIT cells were suspended in 100 μL PBS and were transferred via retroorbital injections into lightly anesthetized Rag1^−/−^ mice. Transferred mice were monitored to confirm recovery following injections and rested for 1 week to allow for MAIT expansion before using the intranasal challenge model. Mice were challenged with an *A. alternata* extract (Greer laboratories Inc., North Carolina, Catalog# NC1620293), 10 μg in 50 μL PBS via intranasal instillation on Days 16, 19, 21 and 23 under anesthesia with isoflurane, while the control mice received intranasal instillation of PBS [60]. Mice were euthanized, and brochoalveolar lavage (BAL) and lungs were collected on Day 23.

### 2.8. BAL Fluid Collection and Analysis

Euthanized animals were placed in the prone position, and a small incision was made to expose the trachea, followed by a 1–3 mM width incision made to the trachea for cannulation. A 1 mL syringe was filled and fitted onto the cannula, and the lungs were slowly flushed three times with 1 mL of Dulbecco’s PBS. The first wash was collected into a separate tube and centrifuged at 300 × *g* for 10 min at 4°C. After centrifugation, the supernatants were separated from cell pellets for cytokine analysis by ELISA. The second and third washes were collected and centrifuged at 300 × *g* to separate cellular content from the supernatants; the cellular pellets from the three washes were combined, and total cells were counted using the TC‐20 automated counter (Biorad) with trypan blue exclusion.

### 2.9. Lung Histology and Scoring

The chest cavity was exposed, and lungs were cleared of blood by cardiac perfusion with a saline solution. The left lung lobe was isolated and prepared using the gentle MACS Lung Dissociation kit (Miltenyi Biotech) according to the manufacturer’s protocol for flow cytometry analysis. The right lung lobe was fixed by tracheal instillation of 10% neutral buffered formalin and kept in 10% formalin overnight. Lungs were then transferred to 70% ethanol. Following paraffin embedding, 5 µm sections were cut and stained with hematoxylin and eosin (H&E) by ARUP, at the University of Utah. About 20x images were acquired using a Zeiss Axioscope 7 (Carl Zeiss Meditec, Inc., Dublin, CA). Inflammation scores were calculated as mentioned in [31], briefly the calculation is as follows: (% of bronchial/bronchiolar epithelium with infiltrate X measured number of cellular depths of peribronchial infiltrate) + (% of pulmonary veins with infiltrate X measured number of cellular depths of perivenous infiltrate). This score was calculated on two sections per animal and three–eight animals per group from two independent experiments.

### 2.10. Estrogen and Cytokine ELISA

Estrogen concentration in patient serum was measured using a 17β‐estradiol high sensitivity ELISA kit (sensitivity 14 pg/mL, cat# ADI‐900‐174, Enzo Life Sciences Inc., Farmingdale, NY) after extraction according to the manufacturer’s protocol. BAL fluids from all groups were centrifuged (400 g) at RT for 10 min to clear cellular debris prior to testing. IFN‐γ, IL‐17, and IL‐13 Duo‐set ELISA (R&D Systems, Minneapolis, MN) was performed according to the manufacturer’s instructions. Absorbance was measured on the SpectraMax M3 (Molecular Devices LLC, San Jose, CA) at 450 nm with a 570 nm wavelength correction.

### 2.11. Statistics

**Figure 2:**
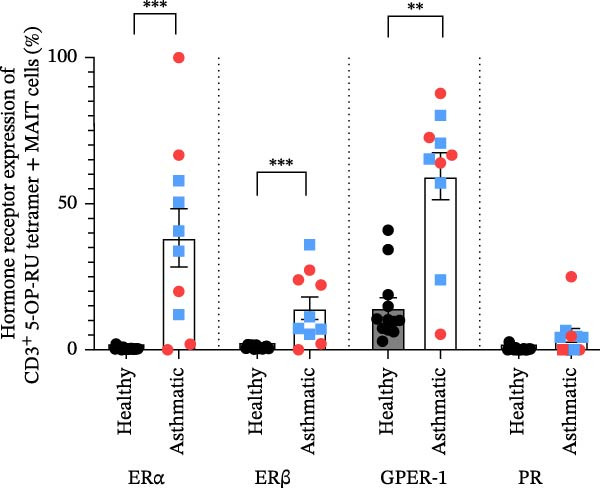
MAIT cells from asthmatic patients express higher levels of estrogen receptors as compared to healthy controls. Expression levels of estrogen receptor alpha (ERα), estrogen receptor beta (ERβ), G‐protein coupled estrogen receptor 1 (GPER‐1) and progesterone receptor (PR) were determined on circulating MAIT cells from healthy subjects and asthmatic patients by flow cytometry. The frequencies of expression in MAIT cells of healthy (*n* = 12) and asthmatics (*n* = 10, blue squares indicate male and red circles indicate female asthmatics) are shown. Data are shown as mean ± standard error of the mean (SEM) from two independent experiments. The Mann–Whitney *U* test was used to determine statistical differences between healthy and asthmatic groups. Statistical significance was assigned as *p*‐value less than 0.05;  ^∗∗^ *p* < 0.01;  ^∗∗∗^ *p* < 0.001.

Statistical analyses were performed using Prism software version 10 (GraphPad). The Wilcoxon matched‐pair signed‐rank test was used for comparison between untreated and estrogen‐treated groups or for comparison between estrogen and estrogen antagonist groups. The Mann–Whitney *U* test was used to determine statistical differences between the healthy and asthmatic groups (Figures 1–4). One‐way ANOVA followed by Holm–Sidak’s multiple comparison test was used to determine statistical differences between multiple groups (in Figure 5). Data presented in the text and figures are means with standard error of the mean (SEM). *p*‐Values corrected for multiple comparisons ≤0.05 were considered significant.  ^∗^ *p* ≤ 0.05,  ^∗∗^ *p*  < 0.01,  ^∗∗∗^ *p*  < 0.001,  ^∗∗∗∗^ *p*  < 0.0001.

Figure 1Circulating MAIT cell frequencies are lower in asthmatic patients compared to healthy individuals. (A) Gating strategy and representative flow cytometry plot of circulating MAIT cells (live CD45^+^ CD3^+^ 5‐OP‐RU tetramer+) in healthy subjects and asthmatic patients. (B) MAIT cells as a percentage of viable CD45^+^ cells. (C) Absolute counts of MAIT cells per mL of blood collected. (D) CD8^+^ MAIT cells as a percentage of total MAIT cells. (E) CD4^+^ MAIT cells as a percentage of total MAIT cells. Blue, square data points indicate male asthmatics, open square points are male healthy controls (*n* = 12 healthy and *n* = 12 asthmatics). Red circle data points indicate female asthmatics, while open circles indicate female healthy controls (*n* = 18 healthy and *n* = 12 asthmatics). Data are representative of the mean ± standard error of the mean of each group from four independent experiments. A Mann–Whitney *U* test was used to determine statistical differences between healthy and asthmatic groups. Statistical significance was assigned when *p*‐value was less than 0.05;  ^∗^ *p* < 0.05,  ^∗∗^ *p* < 0.01.

**Figure:**
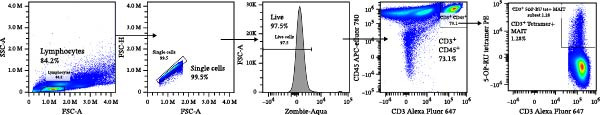
(A)

**Figure:**
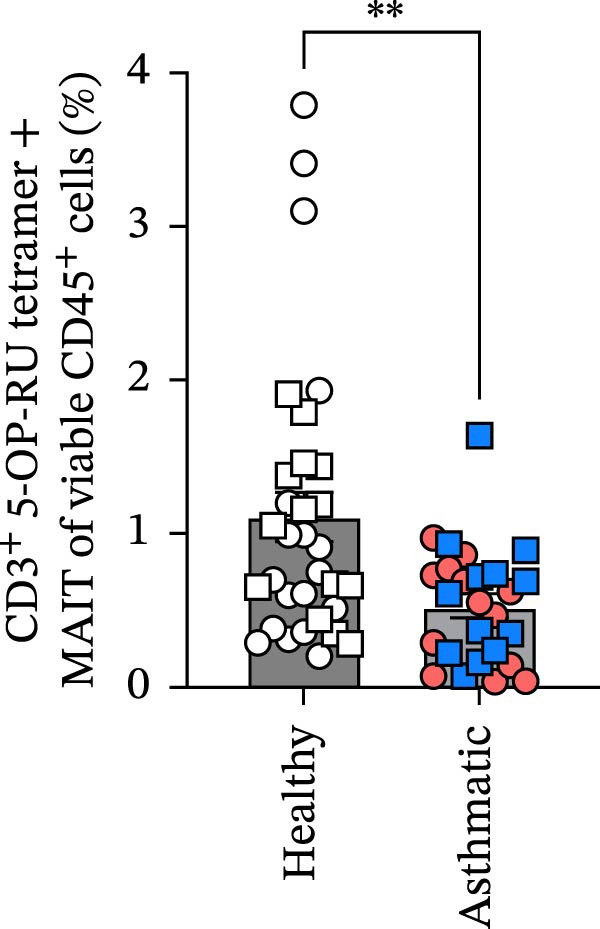
(B)

**Figure:**
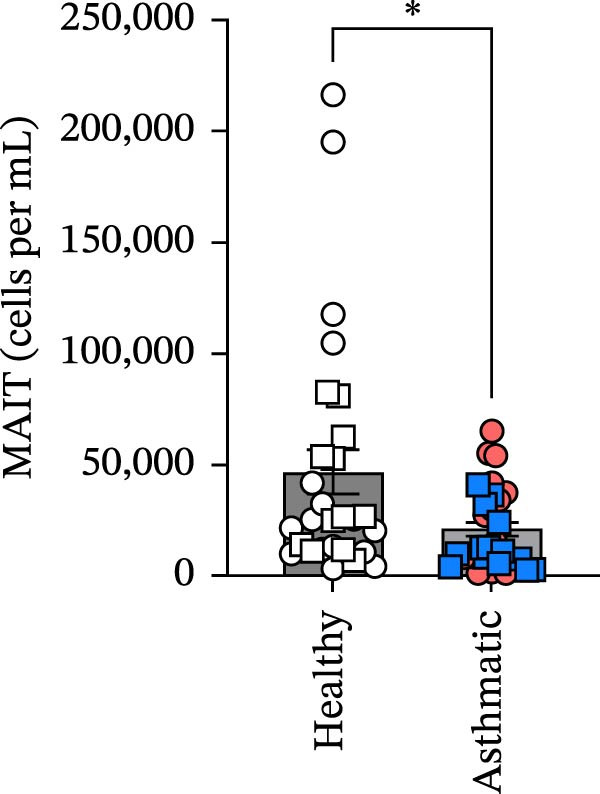
(C)

**Figure:**
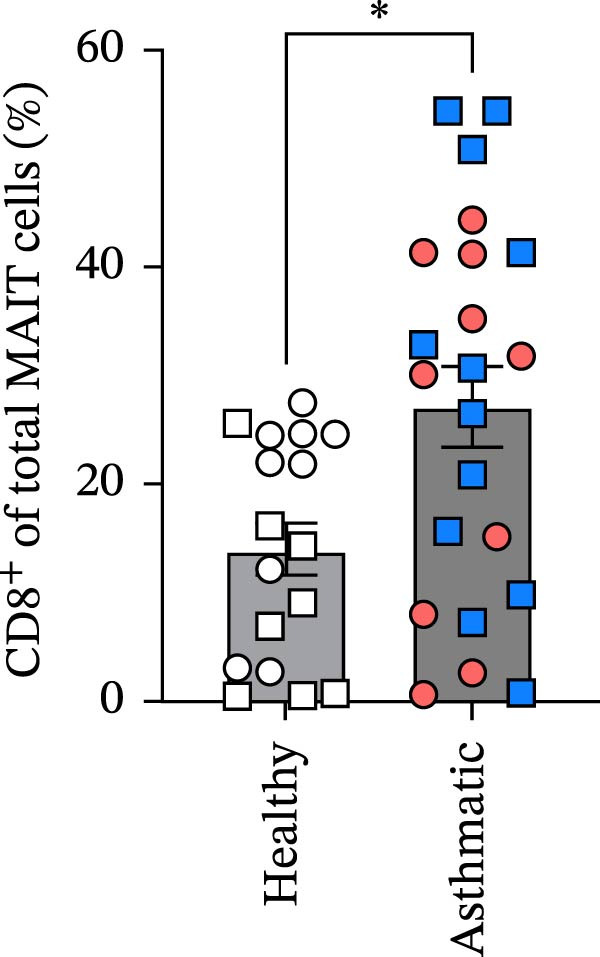
(D)

**Figure:**
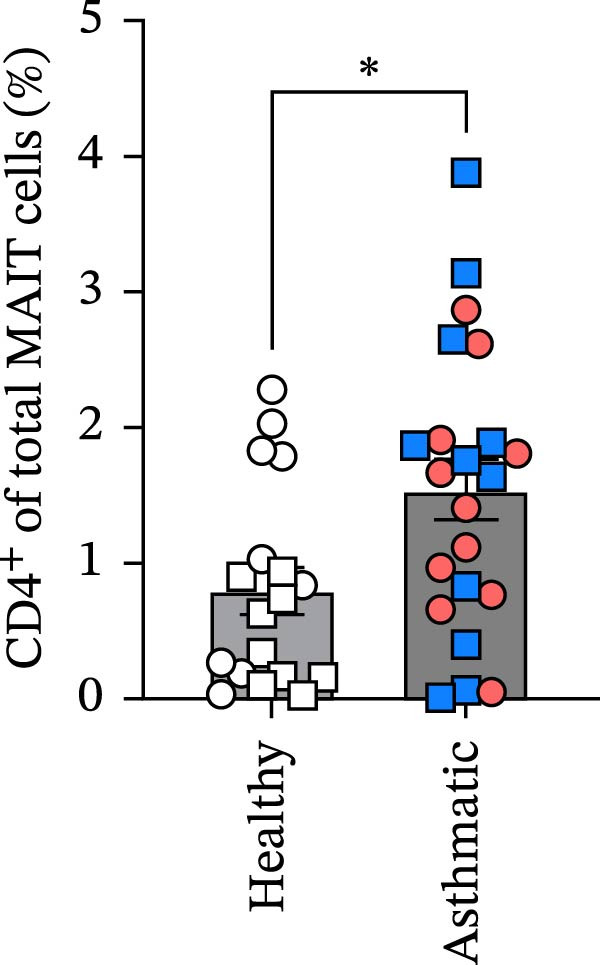
(E)

Figure 3Estrogen treatment decreases MAIT IFN‐γ+ cells in asthmatics compared to healthy individuals and untreated asthmatics. (A) Experimental timeline is shown. PBMCs were treated with estrogen (10 pg/mL; healthy E2 and asthmatic E2) and without estrogen (healthy and asthmatic; Figure 3A) or with and without estrogen + antagonist ICI 182,780 (200 nM) or G36 (1.2 μM; Figure 3D) and cultured for 4–5 days in survival cytokines (IL‐2; 10 ng/mL, IL‐7; 10 ng/mL). After 4–5 days, cells were stimulated with 5‐OP‐RU for 6 h as described in methods. (B) Flow cytometric analysis of IFN‐γ production in MAIT cells of healthy (*n* = 12) and asthmatic individuals (*n* = 10, blue squares indicate male and red circles indicate female asthmatics) with and without estrogen treatment in unstimulated, 5 OP‐RU stimulated PBMCs. (C) Representative flow cytometry plots showing IFN‐γ expression gated on CD3^+^ 5 OP‐RU tetramer+ MAIT cells, numbers indicate IFN‐γ + events as a percentage of MAIT cells. (D) Flow cytometry analysis of IFN‐γ production in MAIT cells of healthy (*n* = 12) and asthmatic individuals (*n* = 10) with estrogen and estrogen antagonists’ treatment in 5 OP‐RU stimulated PBMCs. Data are shown as mean with standard error of mean from 2 to 3 independent experiments. The Wilcoxon matched pair signed rank test was used to compare untreated and estrogen treated groups or to compare estrogen and estrogen antagonist groups. The Mann–Whitney *U* test was used to determine statistical differences between healthy and asthmatic groups. Statistical significance was assigned when *p*‐value was less than 0.05;  ^∗^ *p* < 0.05,  ^∗∗^ *p* < 0.01;  ^∗∗∗^ *p* < 0.001;  ^∗∗∗∗^ *p* < 0.0001; ns, *p* = 0.27.

**Figure:**
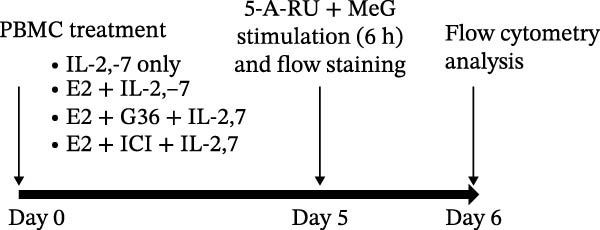
(A)

**Figure:**
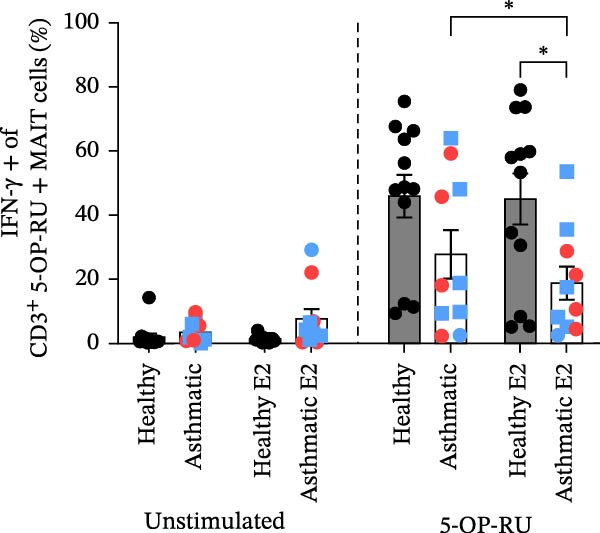
(B)

**Figure:**
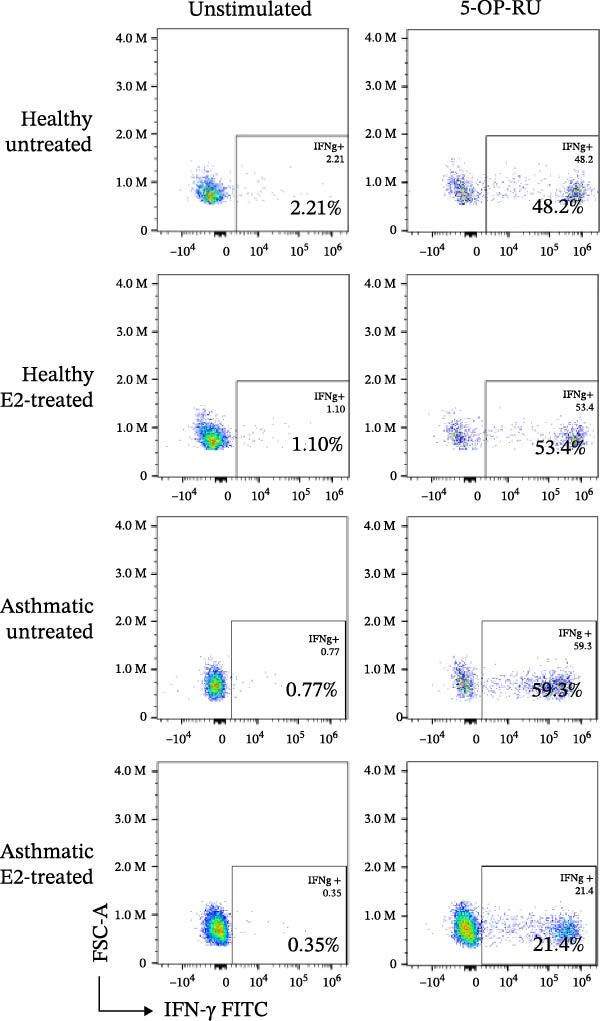
(C)

**Figure:**
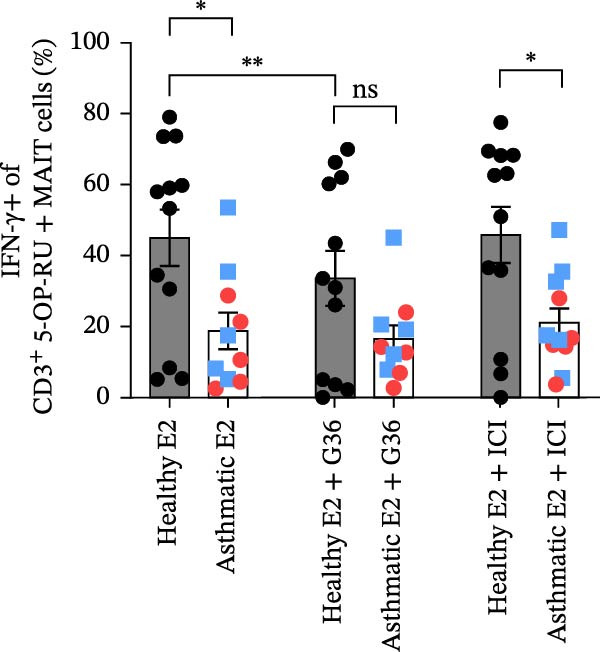
(D)

Figure 4Adoptive transfer of G36‐treated MAIT cells amplifies *Alternaria*‐induced allergic inflammation. (A) An overview of our experimental protocol is shown. In phase 1, MAIT cells are expanded in male and female, C57BL/6 mice using 5‐A‐RU and MeG. In phase 2, MAIT cells were harvested from donor mice then treated overnight with recombinant mouse IL‐12 (10 ng/mL), IL‐15 (10 ng/mL) and IL‐18 (10 ng/mL) in no estrogen, estrogen (10 pg/mL), or with estrogen and G36 (1.2 μM). Cultured MAIT cells were retro‐orbitally transferred into sex‐matched RAG 1^−/−^ recipient mice. In phase 3, RAG1^−/−^ mice were challenged with intranasal *Alternaria* (Alt, 10 μg/mice) at Days 16, 19, 21, and 23. Mice were humanely euthanized, and lungs were inflated then excised for paraffin embedding. (B) Representative images (20x) of H&E staining for each group of animals are shown. (C) Histological scoring was performed on two sections per animal, 3–8 animals per group. Data are shown as mean ± SEM for two independent experiments. The Mann–Whitney *U* test was used to determine statistical differences between MAIT; Alt and MAIT + E2 + G36; Alt groups. Statistical significance was assigned when *p*‐value was less than 0.05;  ^∗^ *p* < 0.05.

**Figure:**
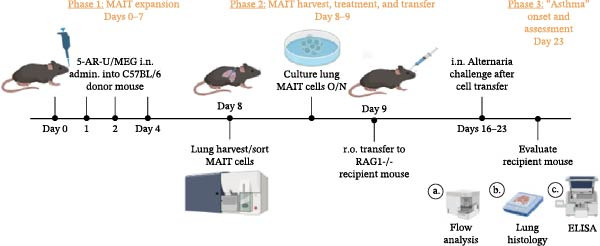
(A)

**Figure:**
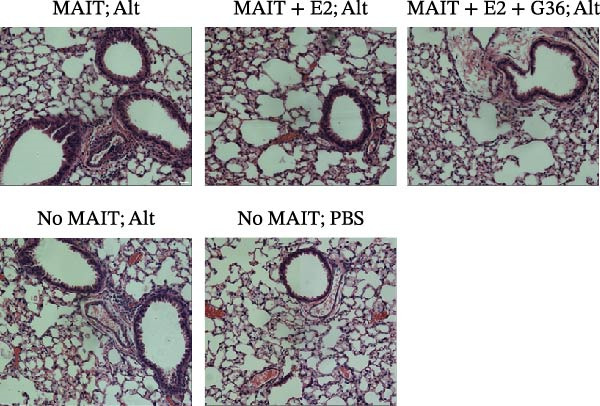
(B)

**Figure:**
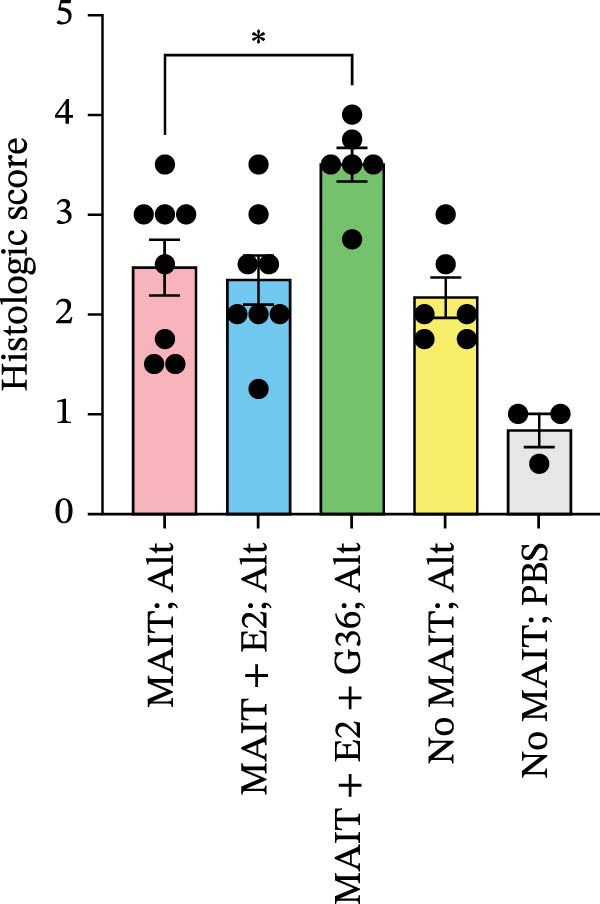
(C)

Figure 5Adoptive transfer of G36‐treated MAIT cells increases eosinophils in comparison to mice that received MAIT cells. Bronchoalveolar lavage fluid was collected from RAG1^−/−^ mice challenged with intranasal *Alternaria* (Alt; 10 μg/mice) and cleared by centrifugation (400 × *g*) prior to analysis by ELISA. (A) IFN‐γ, (B) IL‐17 and (C) IL‐13 proteins are shown as pg/mL of BAL fluid returned following lavage. (D) A representative gating strategy for identifying eosinophils (live singlet CD45^+^ CD11c‐ Siglec‐F+) and neutrophils (live singlet CD11c+ Siglec‐F+ Ly6G+ MHCII^lo^) in BAL is shown. (E) Total eosinophil counts and (G) neutrophil (PMN) counts were determined in BAL by multiplying the percentages of each cell population by the total number of viable cells recovered from lavage. (F) Eosinophils as a percentage of viable CD45^+^ cells, and (H) neutrophils as a percentage of total CD45^+^ cells in the BAL were determined by flow cytometry. Data are shown as mean ± SEM for two independent experiments. One‐way ANOVA followed by Holm–Sidak’s multiple comparison test was used to determine statistical differences between groups. Statistical significance was assigned when *p*‐value was less than 0.05;  ^∗^ *p* < 0.05,  ^∗∗^ *p* < 0.01;  ^∗∗∗^ *p* < 0.001;  ^∗∗∗∗^ *p* < 0.0001.

**Figure:**
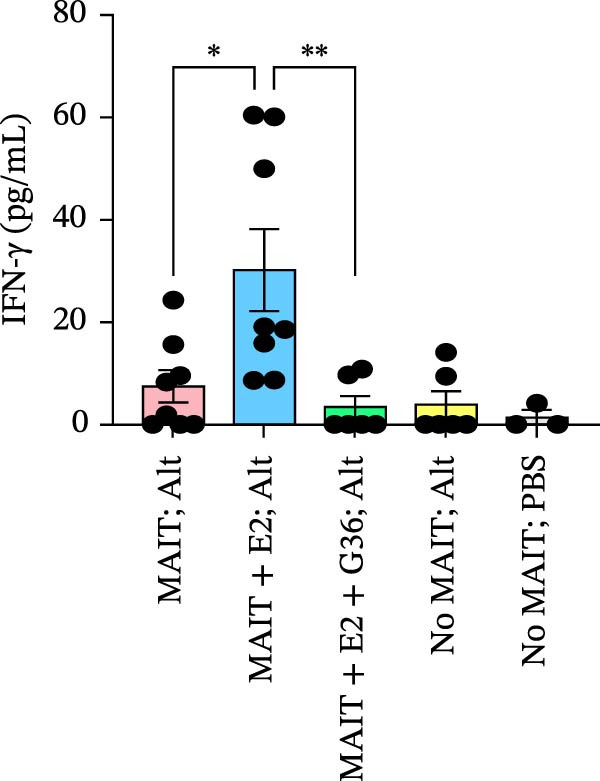
(A)

**Figure:**
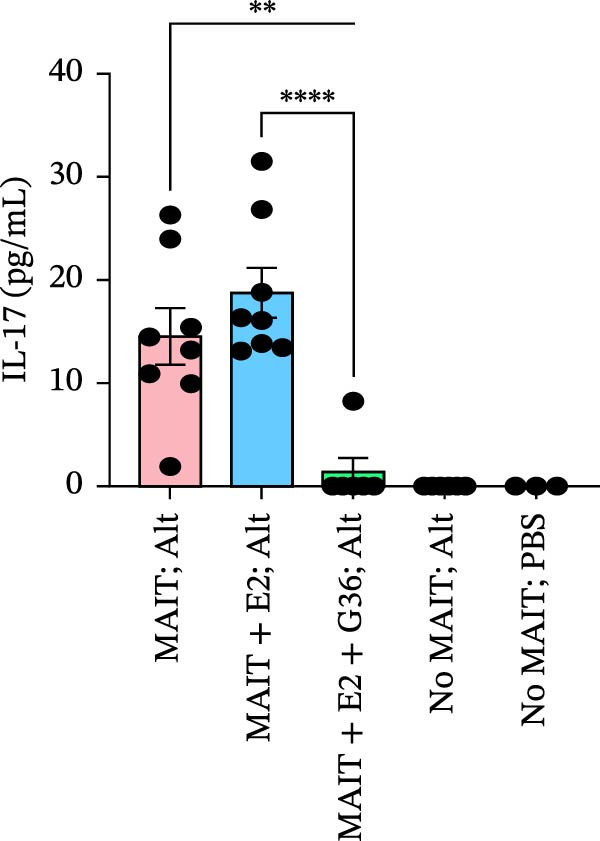
(B)

**Figure:**
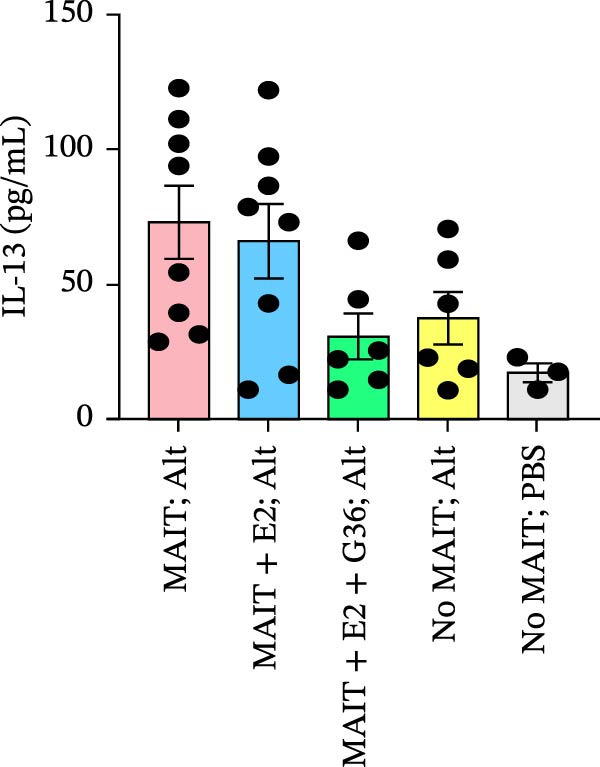
(C)

**Figure:**
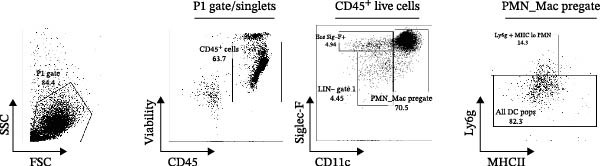
(D)

**Figure:**
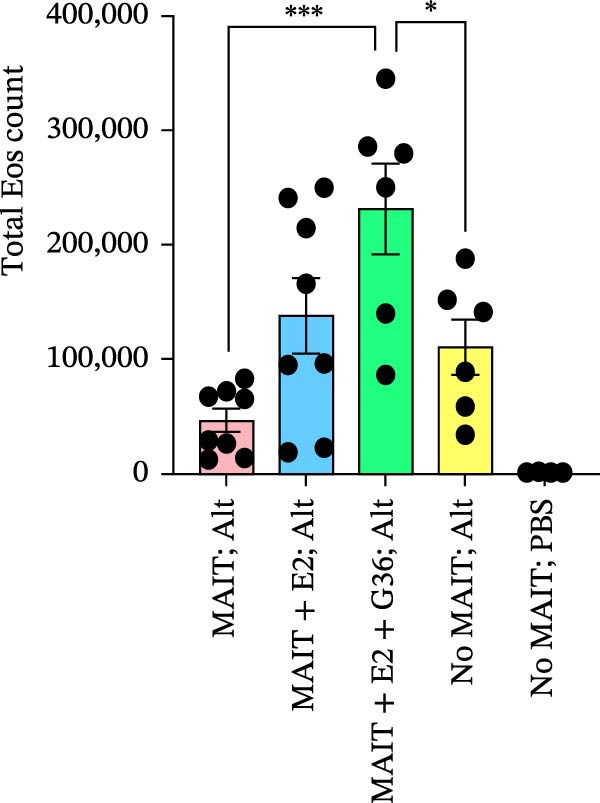
(E)

**Figure:**
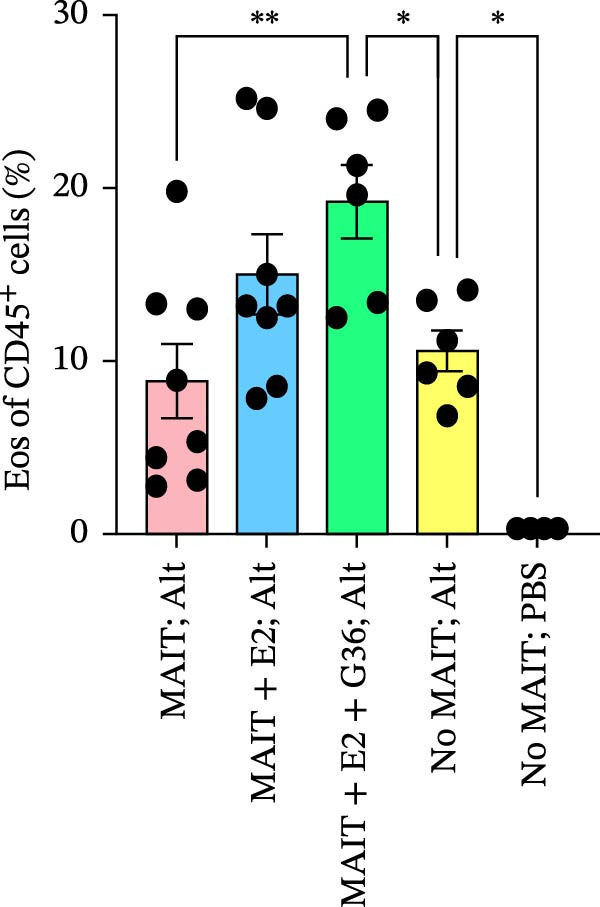
(F)

**Figure:**
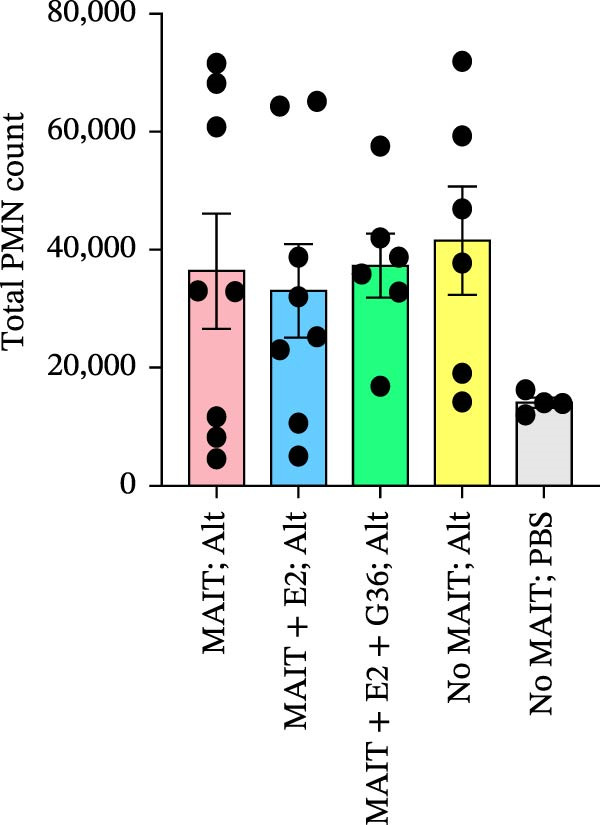
(G)

**Figure:**
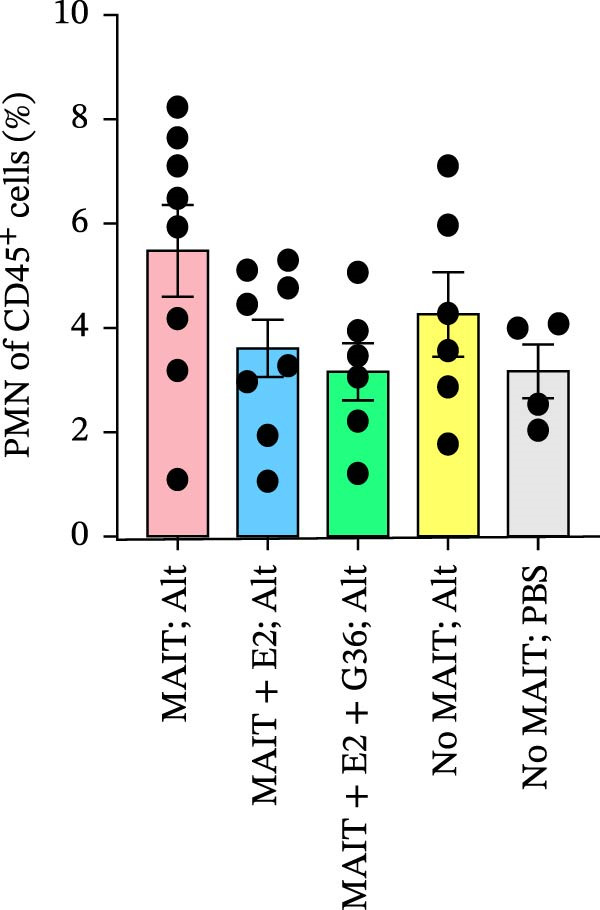
(H)

## 3. Results

### 3.1. Circulating MAIT Cell Frequencies are Lower in Asthmatic Patients Compared to Healthy Individuals

MAIT cells were evaluated in the blood of 30 healthy subjects (12 males and 18 females) and 24 asthmatic participants (12 males and 12 females) (Table 1). MAIT cells were defined as live CD45^+^ CD3^+^ 5‐OP‐RU tetramer^+^ (Figure 1A). We found lower frequencies of MAIT cells among viable CD45^+^ cells and fewer MAIT cells per mL of blood collected in asthmatics compared to healthy individuals (Figure 1B,C). Additional analysis showed that CD8^+^ MAIT cells and CD4^+^ MAIT cells as percentage of total MAIT cells were higher in asthmatics compared to healthy individuals (Figure 1D,E). There were no significant sex differences in MAIT cell percentages or absolute counts in either healthy individuals or asthmatics. In healthy participants, MAIT cell percentages were 1.03 ± 0.14% in males and 1.17 ± 0.27% in females (*p* = 0.65), and MAIT cell counts were 37,853 ± 7910 in males and 53,352 ± 17,034 in females (*p* = 0.41). In asthmatics, MAIT cell percentages were 0.59 ± 0.12% in males and 0.48 ± 0.09% in females (*p* = 0.47), and MAIT cell counts were 15,078 ± 3444 in males and 25,844 ± 5580 in females (*p* = 0.11).

### 3.2. MAIT Cells From Asthmatic Patients Express Higher Levels of ERs Compared to Healthy Individuals

Sex hormones interact with a variety of immune cells through their receptors that are expressed intracellularly (ERα, ERβ, and PR) or on the cell surface (GPER‐1). We measured ERs (ERα, ERβ, and GPER‐1) and the PR using flow cytometry on circulating MAIT cells (CD45^+^ CD3^+^ 5‐OP‐RU tetramer^+^) obtained from healthy and asthmatic participants. We found that MAIT cells expressed more ERα (*p*  < 0.001), ERβ (*p*  < 0.001) and GPER‐1 (*p*  < 0.01) when they were derived from asthmatics compared to healthy individuals (Figure 2). Additionally, the MFI values were higher in asthmatics, as shown in Supporting Information 2: Figure S1. PR expression was relatively low on MAIT cells, and we did not detect a statistical difference in PR expression between healthy and asthmatic individuals. No statistically significant sex differences were detected in MAIT hormone receptor expression; however, the limited sample size may have reduced the power to detect subtle sex‐related effects.

To validate receptor expression, MAIT cells were expanded from healthy human donors and flow‐sorted prior to analysis of *ESR1* and *GPER-1* expression by RT‐PCR. We detected transcripts for *ESR1* and *GPER-1* in sorted MAIT cells (Supporting Information 2: Figure S2A), indicating the presence of ER signaling components. In addition to transcript analysis, we performed Western blotting to assess GPER‐1 protein expression. A band corresponding to GPER‐1 (~44 kDa) was detected in flow‐sorted human MAIT cells, similar to the signal observed in the breast cancer cell line MCF‐7 (positive control), confirming protein‐level expression of GPER‐1 in MAIT cells (Supporting Information 2: Figure S2B,C).

### 3.3. Stimulation With MAIT Ligand and Estrogen Results in Lower IFN‐γ+ MAIT Cells in Asthmatic Participants Compared With Healthy Controls

To assess the impact of estrogen on MAIT cell activation with cytokines and MAIT/MR‐1 ligand 5‐OP‐RU, total PBMCs were cultured with estrogen in the presence or absence of a total ER antagonist (ICI 182 780 at 200 nM, referred as ICI [68–71]) and/or the GPER antagonist (G36 at 1.2 μM [72]) for 4–5 days with the MAIT cell survival cytokine IL‐2 and IL‐7, followed by stimulation with 5‐OP‐RU for 6 h (Figure 3A). Estrogen treatment significantly reduced IFN‐γ production by 5‐OP‐RU‐stimulated MAIT cells isolated from asthmatics (Figure 3B,C). When compared to healthy controls, there were less IFN‐γ+ CD3^+^ MAIT cells in 5‐OP‐RU‐stimulated asthmatic PBMC. Estrogen had no effect on non‐MAIT CD3^+^ T cell IFN‐γ responses to 5‐OP‐RU in healthy individuals or asthma patients (Supporting Information 2: Figure S3). We found that combined treatment with estrogen and GPER‐1 blockade (G36) reduced the number of IFN‐γ‐producing MAIT cells compared to estrogen treatment alone. This effect was only detected, however, in 5‐OP‐RU ligand‐activated MAIT cells from healthy individuals (Figure 3D). Not surprisingly, no differences were observed between estrogen and estrogen with total ER blockade (ICI) treatment in PBMC from healthy or asthmatics donors indicating estrogen was not have off‐target effect in culture. Lastly, stratified analyses revealed no significant correlations between serum estrogen levels and circulating MAIT‐cell frequencies within females, males, healthy donors, asthmatic donors, or the combined cohort (Supporting Information 2: Figure S4), likely because of the limited sample size.

### 3.4. Adoptive Transfer of G36‐Treated MAIT Cells Amplifies *Alternaria*‐Induced Allergic Inflammation

To determine the physiological effect of estrogen on MAIT cells in the allergic inflammation induced by *A. alternata*, we adoptively transferred MAIT cells treated with estrogen in combination with ER antagonists into Rag1^−/−^ mice (Figure 4A). Because of our studies in human MAIT cells showing the effect of G36 in MAIT cells from healthy donors, we hypothesized that blocking GPER‐1 would worsen allergic inflammation in *Alternaria*‐challenged animals because of the reduced IFN‐γ. We found sex differences in IFN‐γ production by cytokine‐activated lung MAIT cells of healthy C57Bl/6 mice, with female mice MAIT cells producing more IFN‐γ than those from male mice. E2 + G36‐treated MAIT cells produced significantly lower IFN‐γ compared to E2‐treated or untreated cytokine‐activated MAIT cells (Supporting Information 2: Figure S5). We confirmed successful transfers and survival of MAIT cells in Rag1^−/−^ mice. After transfer each animal had 0.01%–0.09% MAIT cells as proportion of CD45^+^ cells compared to controls that received saline as a negative cell control (no MAIT, Supporting Information 2: Figure S6). Rag1^−/−^ mice that received MAIT + E2 + G36 had more immune cells infiltrate around airways and pulmonary vessels compared to those receiving MAIT cells only and MAIT + E2 (Figure 4B,C). We next confirmed that MAIT + E2 treated cells induced significantly more IFN‐γ in BAL fluids as compared to all other groups (Figure 5A) and G36 treatment in combination with E2 reduced IFN‐γ production. We also found that IL‐17 was increased in the MAIT cell transfer‐only group and the MAIT + E2 treated group compared to E2 + G36 treated MAIT cells (Figure 5B). IL‐13 production was not significantly different between any of the *Alternaria*‐treated groups (Figure 5C).

An increased number of eosinophils or neutrophils in peripheral blood, sputum, and BAL are a characteristic feature of asthma in humans [73], we next characterized eosinophil and neutrophil levels in BAL by flow cytometry. We found increased counts and frequencies of eosinophils in the BAL of mice receiving MAIT only as compared to MAIT + E2 + G36‐treated cells (Figure 5E,F). Blocking GPER‐1 in MAIT cells significantly increased eosinophils in comparison to *Alternaria*‐treated mice that did not receive any MAIT cell transfers. No significant differences were found between the groups for neutrophils in BAL for any of the mice treated with *Alternaria* extract (Figure 5G,H). Taken together, these data confirm that GPER‐1 on MAIT cells is a physiologically relevant target on MAIT cells that may be pharmacologically activated to control allergic inflammation.

## 4. Discussion

Recent studies have highlighted the potential of human and mouse MAIT cells to modulate asthma outcomes [53, 56, 57, 60]. The prevailing research indicates that stabilizing ovarian hormones significantly reduces the onset of severe asthma in women of reproductive age [18, 74, 75] and in menopausal and postmenopausal women [11–14, 75]. In the present study, we examined the effect of estrogen on MAIT cells in patients with asthma and in an *A. alternata*‐induced allergic inflammation animal model. We report lower frequencies of MAIT cells, higher expression of ERs, and the modulation of MAIT cell function by estrogen in asthmatic individuals compared to healthy controls. We also found that eosinophils, a hallmark feature of high‐type 2 asthma, increased in RAG1^−/−^ animals treated with MAIT cells that had GPER‐1 blockade. The data sheds light on a previously unknown role for GPER‐1 blockade in the initiation of allergic inflammation.

Our results are consistent with several studies that have reported reduced MAIT cell frequencies in asthma patients. For instance, a study by Hinks et al. [56] observed a significant reduction in circulating MAIT cells in severe asthma patients compared to healthy controls. In asthmatic patients, significant deficiency in MAIT cells was associated with serum phytochemically derived vitamin *D*_3_ levels and chronic use of corticosteroids [56]. The patients with asthma who participated in the current study all used inhaled corticosteroids in the form of rescue inhalers, and many were on regular maintenance medication for their asthma. While those individuals did not use rescue inhalers 24 h prior to the single blood draw, it is possible that the functional assays, and ER levels and activation with E2, were diminished because of this corticosteroid use. The development and function of MAIT cells following various pharmacologic treatments certainly warrant further investigation, as rescuing these cells and restoring their numbers in circulation would likely improve asthma for those individuals.

Our finding of increased CD8^+^ and CD4^+^ MAIT cell percentages among the total MAIT cells aligns with other research indicating phenotypic changes in MAIT cells in asthma [57]. MAIT cell frequencies were not significantly affected by sex in the current study, likely because of the limited numbers of males and females. Larger population‑based cohorts have reported clearer sex‑associated patterns. In the Milieu Intérieur study of 1000 adults (500 men and 500 women), MAIT‑cell frequencies were consistently higher in women across all age decades, and active smokers showed reduced numbers of several MAIT cell subsets [38]. Similarly, a cohort of 202 healthy Caucasian adults demonstrated significantly higher MAIT‑cell frequencies in women of reproductive age compared with age‑matched men [37]. In contrast, another study of 202 adults found no sex‑based differences but reported a negative correlation between MAIT‑cell frequency and age [43]. Together, these larger datasets highlight sex‑related differences in MAIT cell frequencies that may not be detectable in smaller cohorts such as ours.

Higher expressions of ERs on MAIT cells were found in patients with asthma compared to healthy controls. This elevated expression of ERs on MAIT cells in asthmatics may explain the differential impact of estrogen antagonists in our healthy versus asthmatic cell culture studies. Our findings reveal a complex and context‐dependent role of ER signaling in MAIT cell function. MAIT cells from asthmatic patients exhibited high expression of ERα, ERβ, and GPER1, yet their IFN‐γ production was suppressed by estrogen without being altered by GPER1 inhibition. In contrast, healthy MAIT cells, which did not show estrogen‐mediated suppression, displayed reduced IFN‐γ production upon G36 treatment. These observations suggest that in the inflammatory milieu of asthma, MAIT cells may exhibit dysregulated GPER‐1 expression or downstream signaling pathways, potentially due to chronic activation. In asthmatics, MAIT cells express high levels of ERα and ERβ in addition to GPER‑1. Thus, even when GPER‑1 is blocked, estrogen may continue to signal through ERα and ERβ, maintaining suppression of IFN‐γ. Prior studies have shown that ERα can contribute to AHR and allergic inflammation, supporting the possibility that these receptors compensate for GPER1 inhibition in asthmatics [76]. We propose that asthmatic MAIT cells exhibit reduced basal GPER‐1 activity despite upregulated receptor expression—a compensatory but functionally insufficient state. In this hypoactive context, antagonism cannot further suppress minimal signaling (hence no effect), while agonism can overcome this dysfunction through receptor resensitization or threshold surpassing. Administration of the GPER‐specific agonist G‐1 has been shown to attenuate airway inflammation in asthmatic mice through IL‐10 [77, 78]. Similar G‐protein‐coupled receptors dysfunction has been well‐documented in chronic airway inflammation, where β2‐adrenergic receptors undergo desensitization through mechanisms like S‐nitrosylation, resulting in uncoupled receptors with diminished signaling despite maintained expression [79]. In contrast, in healthy MAIT cells, GPER‐1 inhibition suppresses IFN‐γ production, suggesting that baseline GPER‐1 signaling supports normal effector function. Future studies examining downstream pathways such as MAPK, PI3K/Akt, and NF‑κB will be important to determine whether the observed effects are specific to G36 inhibition of GPER‑1 or reflect broader modulation of ER signaling.

Estrogen has been shown to modulate the production of IL‐17 in T cells and other innate cells, which are involved in asthma pathogenesis [31, 80–82]. The frequency of IL‐17‐producing MAIT (MAIT‐17) cells was found to be positively correlated with the number of severe exacerbations and asthma symptoms [57, 83]. Although prior work has reported an increase in IL‐17A–producing “Th17‐like” MAIT cells in female patients with ankylosing spondylitis compared with female healthy donors [41], in our study, RAG1^−/−^ mice receiving E2 and the GPER antagonist G36‐treated MAIT cells exhibited markedly reduced IL‐17 levels in the BAL. This suggests that different inflammatory contexts of MAIT activation such as autoimmune inflammation in ankylosing spondylitis versus acute allergic inflammation in our model, can drive distinct MAIT cell response [84].

Our findings indicate that IFN‐γ production by MAIT cells is higher in female mice compared to male mice. This sex‐specific difference in MAIT cell function is consistent with previous observations in other innate‐like T cell subsets, such as invariant natural killer T (iNKT) cells, where females exhibited enhanced IFN‐γ responses [85]. Consistent with our findings, Chen et al. reported that MAIT cells in females exhibit a transcriptional profile enriched for IFN‐γ signaling and antiviral pathways, suggesting a functionally superior phenotype compared to males [40]. Their single‐cell RNA‐seq analysis revealed that females were skewed toward an immune‐active “MAITα” cluster (enriched in several immune process pathways like IFN‐γ signaling and antigen presentation), while males were enriched in a more dysfunctional “MAITβ” cluster (stressed/apoptotic phenotype enriched in cellular responses to external stimuli). Although the influence of sex hormones on MAIT cells remains less well defined, prior studies have shown that estrogen can modulate immune responses, including cytokine production, in T cells and innate lymphocytes [85–87]. Mechanisms accounting for gender dimorphism in cytokine‐stimulated MAIT cell immune responses are poorly understood and warrant further investigation.

Understanding the mechanisms by which estrogen influences asthma is of paramount importance and could inform the management of female asthmatics. In healthy donors, IFN‐γ responses were attenuated by G36 (a GPER1 antagonist), whereas similar attenuation was not observed with ICI 182,780 (Fulvestrant), a classical ER (ERα/ERβ) antagonist. This suggests that the effects of E2 in our system are likely mediated predominantly through nonclassical estrogen signaling pathways, specifically via GPER1, rather than through classical nuclear ERs (ERα or ERβ), which are targeted by ICI 182,780. Even though ICI is an antagonist of classic receptors, it is a GPER agonist [88, 89], and the related signaling pathways are still not fully understood. Studies have shown that G36 selectively blocks GPER1 signaling without affecting ERα or Erβ [72], making it a more precise tool for dissecting GPER1‐mediated effects. Here, we demonstrated that the treatment of MAIT cells with G36 increased eosinophil response to allergen in mice. Our findings of exacerbated allergic inflammation upon transfer of G36‐treated MAIT cells are consistent with research demonstrating the role of GPER‐1 in regulating immune responses [90]. Studies have shown that GPER‐1 antagonism can exacerbate inflammation [91], and that administration of a GPER‐1 agonist during the allergen challenge phase inhibits allergic airway response in mice [77]. The finding that adoptive transfer of estrogen‐treated MAIT cells to RAG1^−/−^ mice increased IFN‐γ, and that G36 treatment abrogates this effect, is of particular importance because, although generally recognized as pro‐inflammatory, IFN‐γ has been intricately linked to asthma protection in *A. alternata* allergic inflammation model. A likely mechanism involves IFN‐γ‐induced suppression of ILC2 activity that limits asthmatic airway inflammation. It has been shown that MAIT cells play a crucial role in suppressing the cytokine‐producing capacity and proliferation of ILC2 via IFN‐γ production, leading to a decrease in allergic airway inflammation [53, 60]. The increased eosinophil recruitment observed in mice that received G36‐treated MAIT cells aligns with the known role of eosinophils in asthma pathology [92]. IL‐4 and IL‐13 are necessary for the development of airway eosinophilia and IL‐17A is a key driver of neutrophilic asthma in response to *A. alternata* [93], and the lack of significant changes in IL‐13 and neutrophil levels indicates that the primary effect of G36 treatment may be more related to specific inflammatory pathways rather than general cytokine production in MAIT cells. G36 selectively inhibits estrogen‐mediated activation of PI3K by GPER‐1 [72], and it is possible that G36 treatment inhibits the IL‐2/IL‐15R‐mTORC1‐T‐bet axis, linked to IFN‐γ secreting MAIT1 cells differentiation [94], which is known to suppress type 2 inflammation and mucin production and may contribute to airway epithelial repair in eosinophilic inflammation [95]. A plausible explanation for the observation of increased eosinophilia in the absence of elevated BAL IL‐13 levels is that eosinophil recruitment can occur through IL‐5‐dependent mechanisms independent of local IL‐13. Studies show that MAIT cells can produce IL‐5 upon activation [96], which directly promotes eosinophil survival and chemotaxis. Evidence supporting the role of GPER‐1 signaling in MAIT cells in patients with asthma had not been investigated previously. Our findings demonstrate that G36 treated MAIT cells can prevent the development of airway inflammation, suggesting a prophylactic role. The role that hormone‐treated MAIT cells play in asthma pathogenesis and whether these cells can be harnessed prophylactically or therapeutically to reduce asthma burden needs further investigation.

This study has several limitations. First, we did not obtain BAL, sputum, or lung tissue samples from patients. Peripheral blood in human subjects only partially informs of the phenotypic differences in the lung environment between healthy and asthmatic people and likely only in cases where the lung inflammation is high enough to spill over into circulation, leading to bone marrow hematopoiesis. This limitation may contribute to the observed discrepancy in the effect of estrogen on MAIT cells: in mice, estrogen treatment of lung MAIT cells increased IFN‐γ levels in BAL after allergen challenge, while in asthmatic patients, estrogen treatment of peripheral MAIT cells decreased IFN‐γ levels. Recent findings have indicated high heterogeneity of both MAIT cell phenotypes and subsets between blood and tissues as well as between tissues themselves [97, 98]. Second, the blood samples were collected upon study enrollment but prior to treatments with hormones or corticosteroids, that may have greater than 24 h effects, and menstrual cycle‐related hormone fluctuations in progesterone and estrogen in women, and the unknown levels of testosterone in the men, complicates the interpretation of the clinical data in the study population. It will be interesting to study hormone regulation in MAIT cells in a longitudinal study that includes separate phases of the menstrual cycle. In addition, our cohort was predominantly allergic and moderate‐to‐severe asthma patients, and analyses by asthma subtype were limited by small sample size; future longitudinal studies with larger, diverse cohorts will be needed to address this. Third, we did not evaluate GPER1 expression in murine MAIT cells nor utilize GPER1‐deficient mice or GPER1‐specific agonists in this study. Future studies are needed to clarify the role of GPER1 in MAIT cell‐mediated responses during asthma. Lastly, the mechanism by which estrogen acts to influence MAIT cell development and function remains unclear. In this study, we did not examine the independent effects of ERα or ERβ on MAIT cells. In T cells, estrogen can certainly regulate pathways through either of these ERs bringing about diverse T cell repertoires; exploring these with a single‐cell sequencing approach is warranted.

## 5. Conclusions

In summary, this study sheds light on the previously unreported role of estrogen‐induced regulation of MAIT cells in allergic asthma. This study showed higher expression of ERs, and the modulation of MAIT cell function by estrogen in asthmatics compared to healthy individuals. Furthermore, the study also showed the exacerbation of allergic inflammation by G36‐treated MAIT cells, indicating that MAIT cells may play a protective role against *A. alternata*‐induced allergic asthma. It also highlights the potential role of GPER‐1 in MAIT cells as a novel prophylactic target for preventing asthma development, though its therapeutic efficacy in established disease warrants further investigation. Given the rise in the number of patients undergoing treatment with sex hormones and sex hormone modulators for a variety of conditions, in vivo mouse experiments, in conjunction with ex‐vivo studies from asthmatic patient samples, will not only increase the understanding of hormone regulation in MAIT cells but will also open new avenues to explore the protective role of MAIT cells in allergic asthma.

## Author Contributions

Shubhanshi Trivedi designed and performed experiments, analyzed data, and wrote the manuscript. Samuel E. Aamodt, Thomas P. Huecksteadt, and Elizabeth J. Myers performed experiments and provided input on interpretation. Jackson G. Cacioppo and Jeffrey Aubé provided reagents and research interpretation. Robert Paine provided input to research interpretation and edited the manuscript. Daniel T. Leung provided input to the research design and interpretation and edited the manuscript. Kristi J. Warren conceptualized the research, directed the study, analyzed data, and edited the manuscript.

## Funding

The study was supported by the Department of Veteran Affairs (Grants IK2 BX4004219 and 5I01 BX004637) and the Margolis grant awarded to Kristi J. Warren. The University of Utah flow cytometry core is supported by the Office of the Director of the National Institute of Health (Grant S10OD026959) and the NCI (Grant 5P30CA042014‐24).

## Ethics Statement

All protocols were approved by the appropriate IACUC and research advisory committees.

## Consent

All the authors agree with the content of this study and consent for publication.

## Conflicts of Interest

The authors declare no conflicts of interest.

## Supporting Information

Additional supporting information can be found online in the Supporting Information section.

## Supporting information


**Supporting Information 1** The following Supporting Information are available with this article: Graphical Abstract: Model depicting GPER‐1–mediated signaling in human MAIT cells under healthy and asthmatic conditions. The schematic also illustrates how GPER‐1 blockade with G36 in MAIT cell increased A. alternata‐induced inflammation in mice.

**Supporting Information 2** Figure S1: Increased Median fluorescent intensity (MFI) of GPER‐1 receptor expression in asthmatic MAIT cells compared to healthy individuals. Figure S2: GPER‐1 RT‐PCR and western blot analysis in MAIT cells. Figure S3: Estrogen treatment has no effect on IFN‐γ+ T cells in asthmatics and healthy individuals. Figure S4: Correlation between estrogen levels and MAIT cells. Figure S5: Blocking GPER‐1 with G36 suppressed MAIT cell IFN‐γ production in lungs of healthy mice. Figure S6: Percentages of MAIT cell in BAL of RAG1^−/−^ mice after adoptive transfer.

## Data Availability

The data that support the findings of this study are available in the main text and Supporting Information of this article, raw FACS files are available from the corresponding author upon reasonable request.
