# Supplementary figures and images for: Estrogen Induced Regulation of Mucosal‐Associated Invariant T Cells in Asthma
Source: J Immunol Res. 2026 Mar 20;2026:8201923. doi: 10.1155/jimr/8201923 (PMC13140411; doi:10.1155/jimr/8201923)

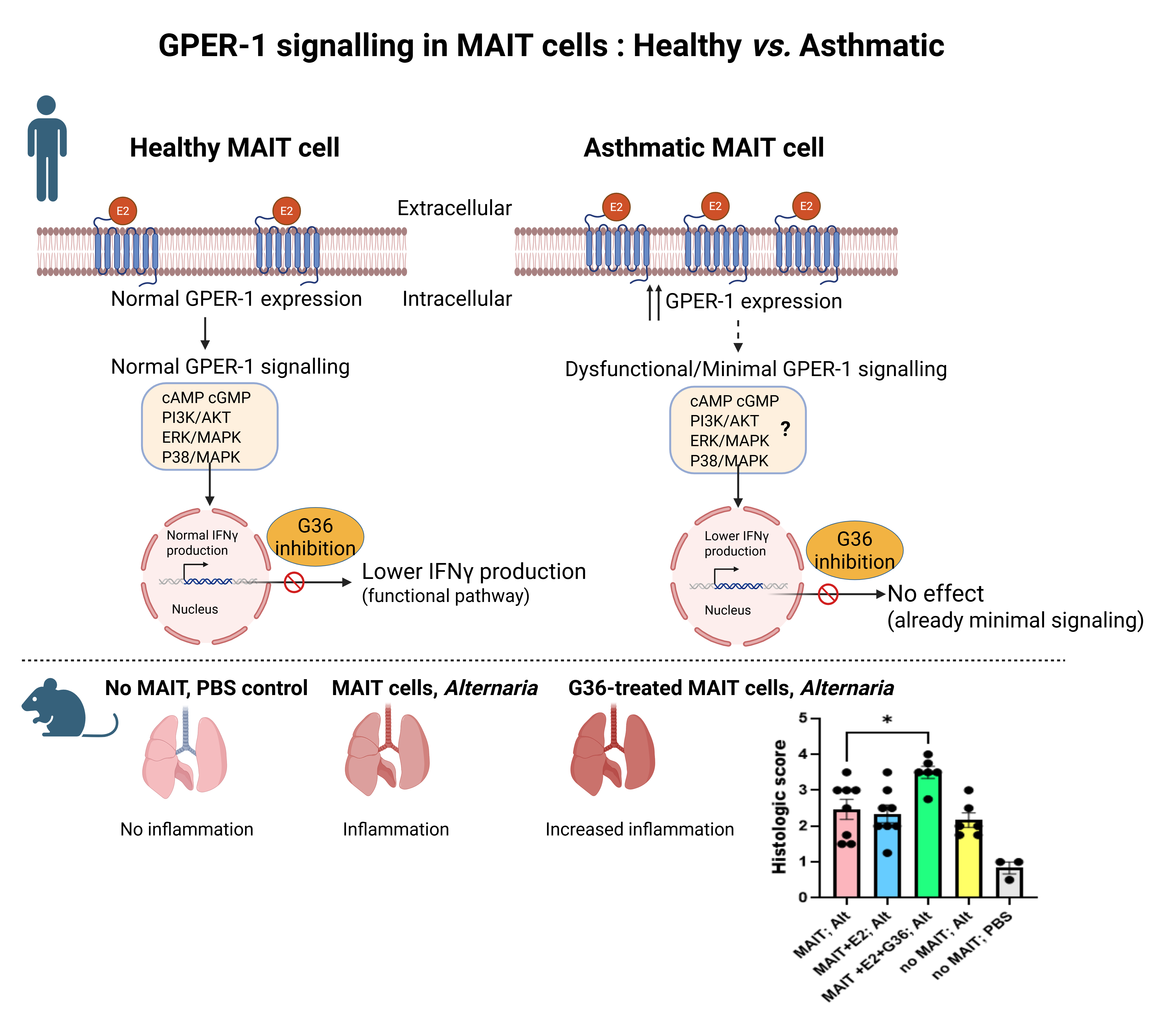

Supplement: Supplementary file 1 — Supporting Information 1 The following Supporting Information are available with this article: Graphical Abstract: Model depicting GPER‐1–mediated signaling in human MAIT cells under healthy and asthmatic conditions. The schematic also illustrates how GPER‐1 blockade with G36 in MAIT cell increased A. alternata‐induced inflammation in mice. [file JIMR-2026-8201923-s001.png]
