# Supplementary material for: Estrogen Induced Regulation of Mucosal‐Associated Invariant T Cells in Asthma
Source: J Immunol Res. 2026 Mar 20;2026:8201923. doi: 10.1155/jimr/8201923 (PMC13140411; doi:10.1155/jimr/8201923)
Supplement: Supplementary file 2 — Supporting Information 2 Figure S1: Increased Median fluorescent intensity (MFI) of GPER‐1 receptor expression in asthmatic MAIT cells compared to healthy individuals. Figure S2: GPER‐1 RT‐PCR and western blot analysis in MAIT cells. Figure S3: Estrogen treatment has no effect on IFN‐γ+ T cells in asthmatics and healthy individuals. Figure S4: Correlation between estrogen levels and MAIT cells. Figure S5: Blocking GPER‐1 with G36 suppressed MAIT cell IFN‐γ production in lungs of healthy mice. Figure S6: Percentages of MAIT cell in BAL of RAG1−/− mice after adoptive transfer. [file JIMR-2026-8201923-s002.pdf]

Supplementary figure 1

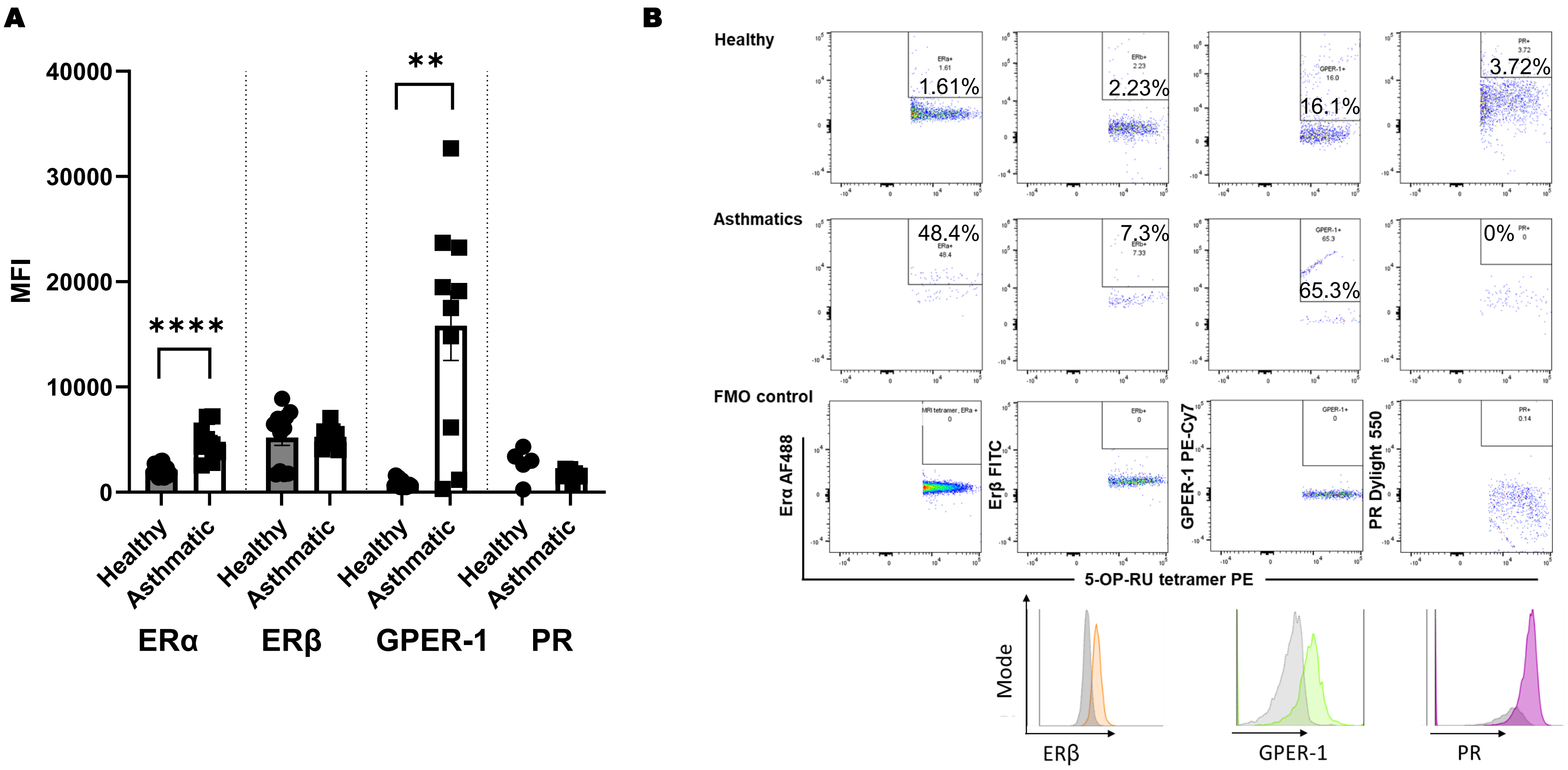

Supplementary Figure 1. Increased Median fluorescent intensity (MFI) of GPER-1 receptor expression in asthmatic MAIT cells compared to healthy individuals. (A) MFI of estrogen and progesterone hormone receptor expression in MAIT cells of H (6 males and 6 females) and A (5 males and 5 females) is shown. (B) Representative flow cytometry plots are shown in which numbers indicate the frequency of flow cytometry events gated on CD3+ 5 OP-RU+ MAIT cells, fluorescence minus one (FMO) controls were used to set gates for fully stained sample). Data are shown as mean with standard error of mean from 3 independent experiments. Mann-Whitney U test was used to determine statistical differences between healthy and asthmatic group. Statistical significance was assigned when p-value was less than 0.05; **$p < 0.01$; ****$p < 0.0001$.

Supplementary figure 2

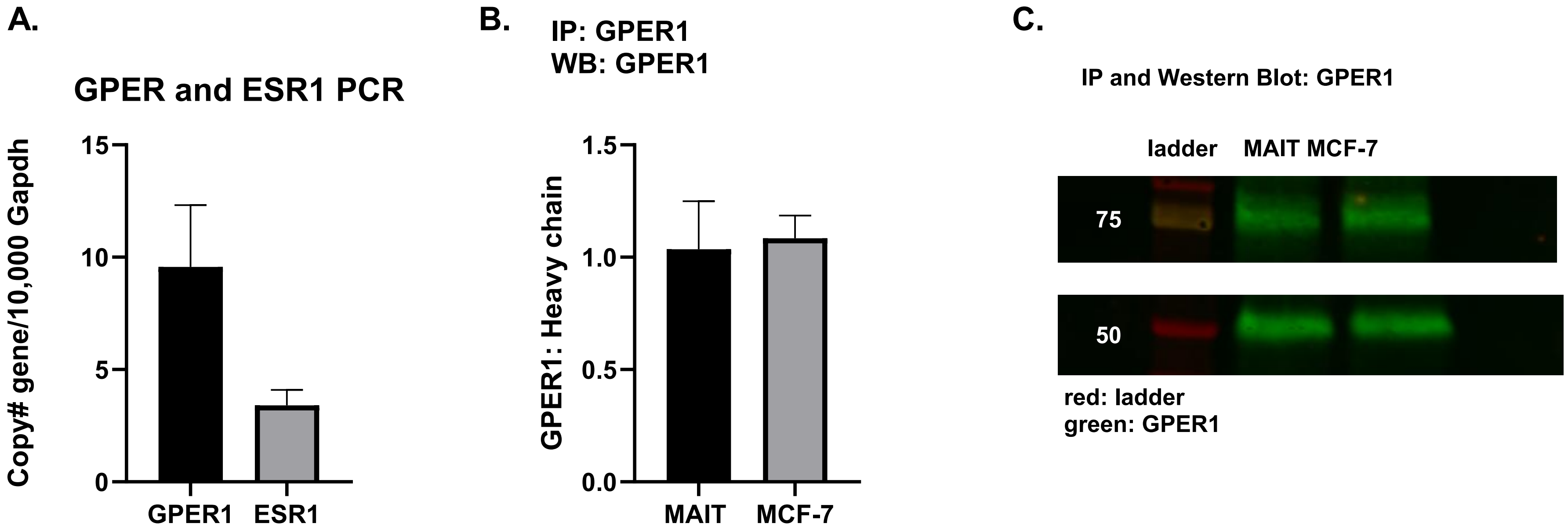

Supplementary Figure 2. GPER-1 RT-PCR and western blot analysis in MAIT cells. (A) GPER and ESR1 RT-PCR analysis in sorted MAIT cells. Samples represent pooled cells from four donors each. Expression was quantified using TaqMan primers (Thermo Fisher Scientific), and copy number was calculated relative to the *Gapdh* housekeeping gene by the ΔΔCT method (B) Quantitation of GPER1 in MAIT and MCF-7 cells in western blot after IP as normalized to the heavy chain protein. N=2. Quantitation was done in ImageJ. (C) Representative Western blot image of GPER and the heavy chain in sorted human MAIT cells (lane 2) and MCF-7 cells (lane 3). N=2. 20 ug of protein was used for immunoprecipitation from MAIT cells (6 donors pooled) and MCF-7 cells. Image was acquired on a Licor Odyssey CLx system.

Supplementary figure 3

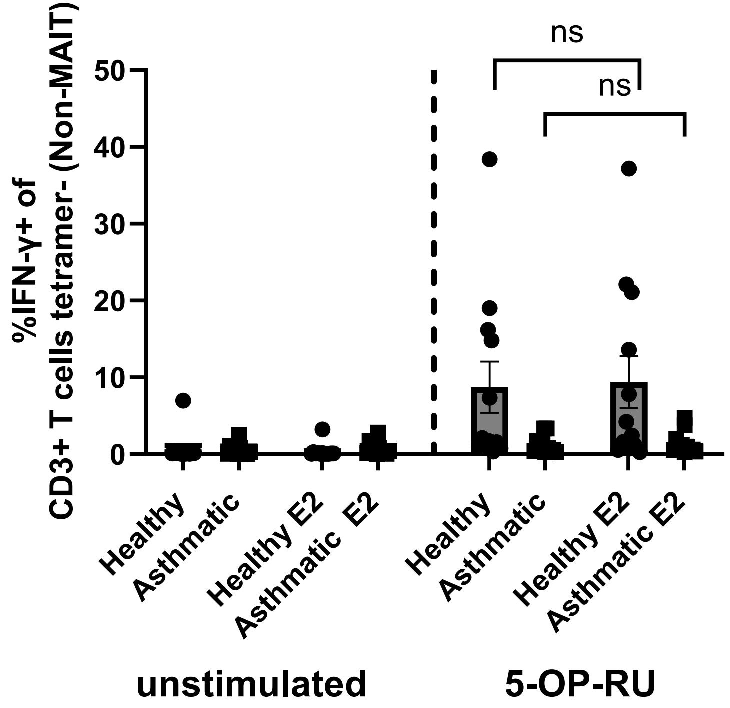

Supplementary Figure 3. Estrogen treatment has no effect on IFN-γ+ T cells in asthmatics and healthy individuals. PBMCs were treated with estrogen (10 pg/ml) (Healthy E2 and Asthmatic E2) and without estrogen and cultured for 4-5 days in survival cytokines (IL-2; 10 ng/ml, IL-7; 10 ng/ml). After 4-5 days, cells were stimulated with 5-OP-RU for 6 hours as described in methods. IFN-γ producing Non MAIT cells (live CD45+ CD3+ 5-OP-RU tetramer -) were analyzed using flow cytometry. The Mann-Whitney *U* test was used to determine statistical differences between the healthy and asthmatic groups.

Supplementary figure 4

A

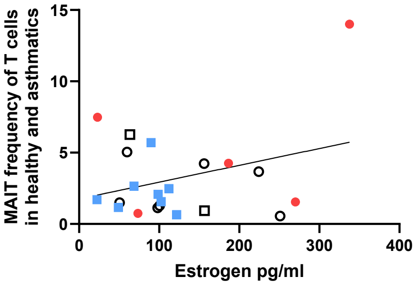

	Estrogen pg/ml vs. MAIT frequency of T cells
Pearson r	
r	0.3154
95% confidence interval	-0.1225 to 0.6505
R squared	0.09945
P value	
P (two-tailed)	0.1528
P value summary	ns
Significant? (alpha = 0.05)	No
Number of XY Pairs	22

B

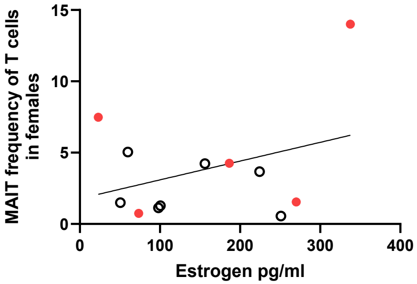

	Estrogen pg/ml vs. MAIT frequency of T cells
Pearson r	
r	0.3441
95% confidence interval	-0.2864 to 0.7666
R squared	0.1184
P value	
P (two-tailed)	0.2735
P value summary	ns
Significant? (alpha = 0.05)	No
Number of XY Pairs	12

C

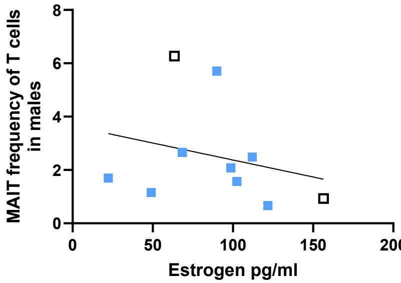

	Estrogen pg/ml vs. MAIT frequency of T cells
Pearson r	
r	-0.2551
95% confidence interval	-0.7623 to 0.4462
R squared	0.06506
P value	
P (two-tailed)	0.4770
P value summary	ns
Significant? (alpha = 0.05)	No
Number of XY Pairs	10

D

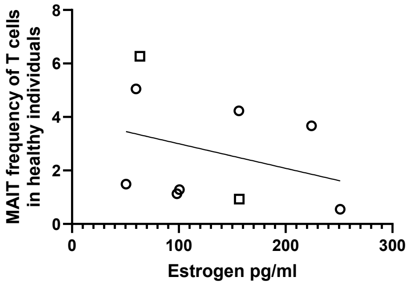

	Estrogen pg/ml vs. MAIT frequency of T cells
Pearson r	
r	-0.3185
95% confidence interval	-0.8111 to 0.4383
R squared	0.1014
P value	
P (two-tailed)	0.4035
P value summary	ns
Significant? (alpha = 0.05)	No
Number of XY Pairs	9

E

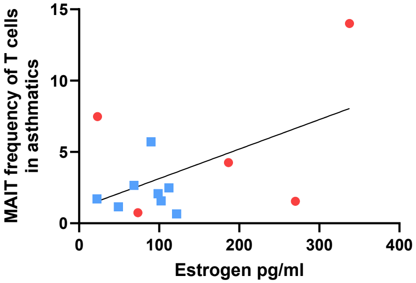

	Estrogen pg/ml vs. MAIT frequency of T cells
Pearson r	
r	0.5180
95% confidence interval	-0.04621 to 0.8316
R squared	0.2683
P value	
P (two-tailed)	0.0698
P value summary	ns
Significant? (alpha = 0.05)	No
Number of XY Pairs	13

Supplementary Figure 4. Correlation between estrogen levels and MAIT cells. A scatter plot showing the correlation between estrogen levels (x-axis) and the count of MAIT cells (y-axis) in (A) a sample of 22 healthy and asthmatic individuals (open circle indicates healthy females, red circle indicates asthmatic females, open square indicates healthy males and blue square indicates asthmatic males), (B) all females, (C) all males, (D) all healthy individuals and (E) all asthmatics. Each point represents an individual sample. The line represents the best fit linear regression line. Pearson correlation coefficient (r) and p-value are reported in a table below the plot to indicate the strength and significance of the correlation.

Supplementary figure 5

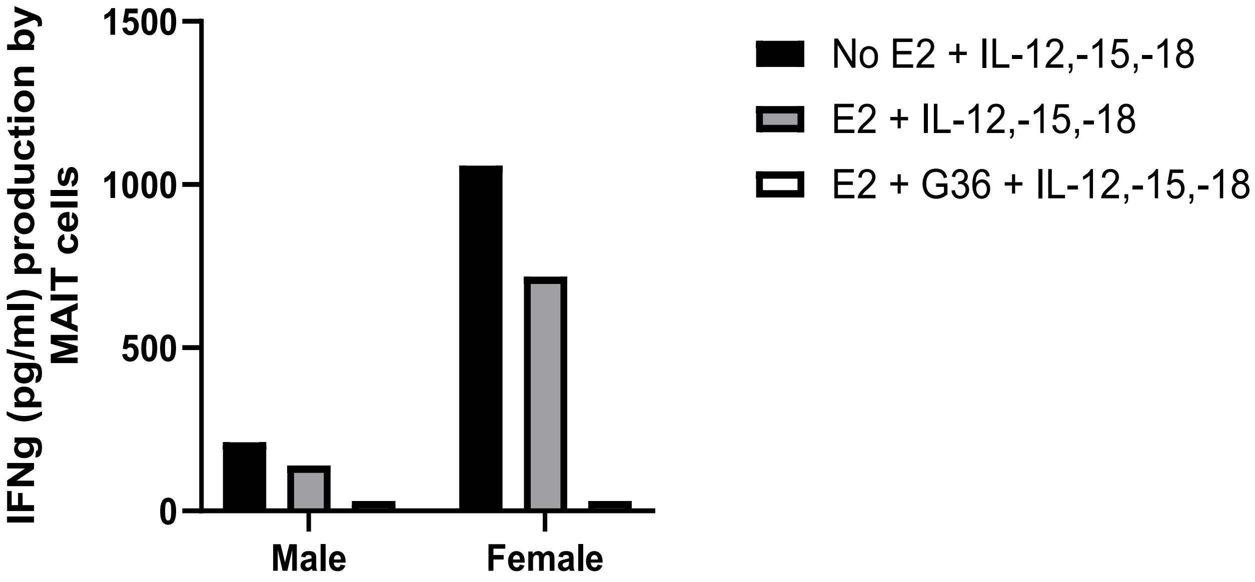

Supplementary Figure 5. Blocking GPER-1 with G36 suppressed MAIT cell IFN-γ production in lungs of healthy mice. MAIT cells were expanded *in vivo* with i.n. 5-OP-RU and MEG in Bl/6 male and female mice (n = 5-10), then flow sorted using CD3 and MR-1 5-OP-RU tetramer (as in methods). Lung MAIT cells were pooled from male and female mice and cultured with E2 (10 pg/ml) and E2 with G36 (1.2μM) in presence of IL-12, -15, -18 cytokines for overnight and IFN-γ was measured in supernatants using ELISA. Data shown is representative of two independent experiments using pooled cells from the lungs of 5-10 mice.

Supplementary figure 6

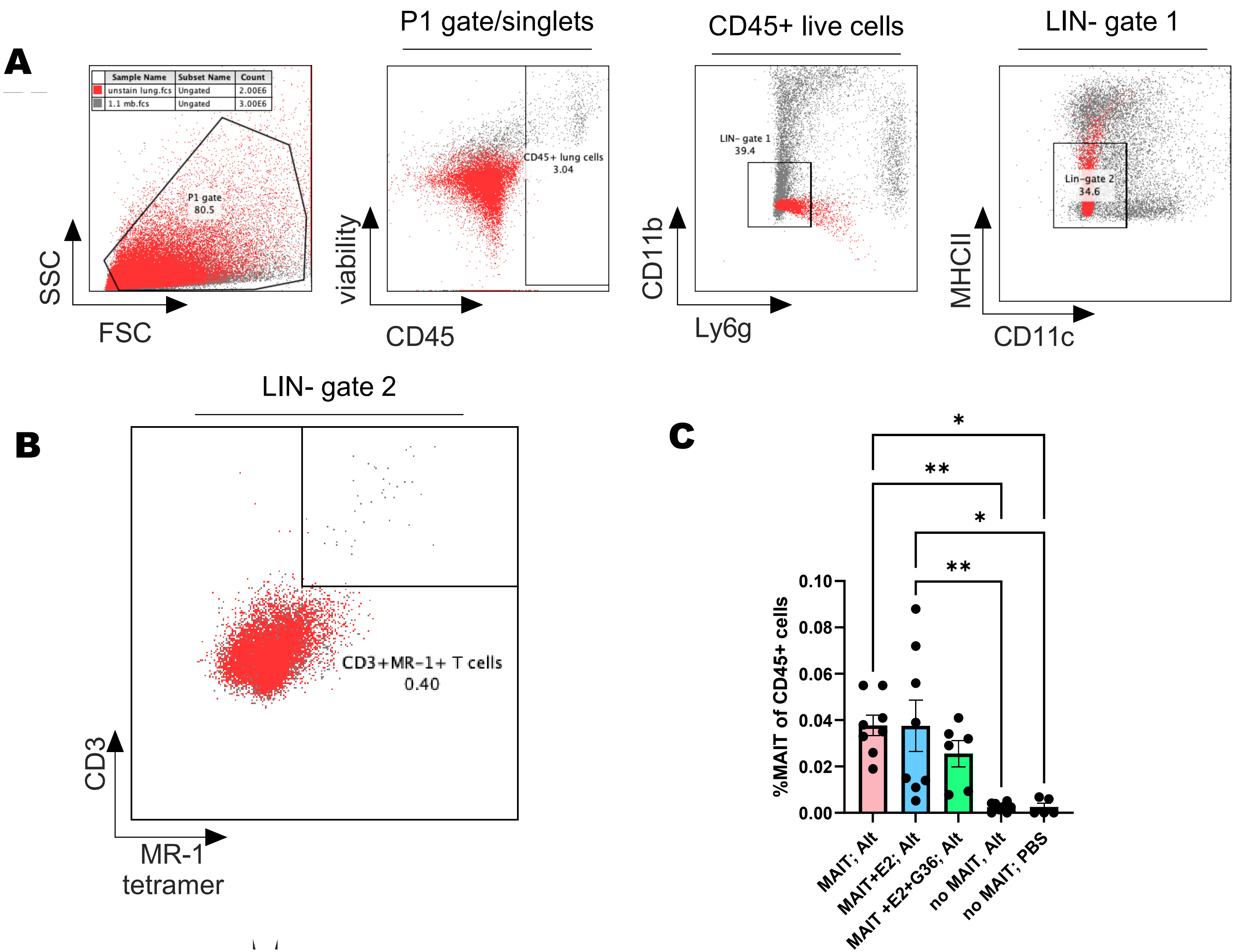

Supplementary Figure 6: Percentages of MAIT cell in BAL of RAG1-/- mice after adoptive transfer. Estrogen treated or untreated MAIT cells were retro-orbitally transferred into RAG1-/- mice and two weeks after transfer BAL was obtained from all groups. A and B show flow cytometry gating strategy of MAIT cells defined as live singlet CD45+ CD11b- Ly6G- MHCII- CD11c- CD3+ MR1 tetramer +. (C) MAIT cells as percentage of CD45+ cells in BAL. The data are shown as mean ± SEM and are representative of two independent experiments. As before, one-way ANOVA followed by Holm-Sidak's multiple comparison test was used to determine statistical differences between groups. Statistical significance was assigned when *p*-value was less than 0.05; $*p < 0.05$, $**p < 0.01$.
